# The regulatory effect of microRNA-21a-3p on the promotion of telocyte angiogenesis mediated by PI3K (p110α)/AKT/mTOR in LPS induced mice ARDS

**DOI:** 10.1186/s12967-019-02168-z

**Published:** 2019-12-26

**Authors:** Yile Zhou, Yajie Yang, Tao Liang, Yan Hu, Haihong Tang, Dongli Song, Hao Fang

**Affiliations:** 1 Department of Anaesthesiology, Jinshan Hospital, Fudan University, 1508 Longhang Road, Shanghai, 201508 People’s Republic of China; 2 Department of Anaesthesiology, Zhongshan Hospital, Fudan University, 180 Fenglin Road, Shanghai, 200032 People’s Republic of China; 3 Zhongshan Hospital Institute for Clinical Science, Shanghai Institute of Clinical Bioinformatics, Shanghai Engineering Research for AI Technology for Cardiopulmonary Diseases, Shanghai Medical College, Fudan University, Shanghai, China; 4 Department of Anaesthesiology, Minhang Branch, Zhongshan Hospital, Fudan University, 170 Xinsong Road, Shanghai, 201199 People’s Republic of China

**Keywords:** Telocyte, miRNA-21a-3p, Angiogenesis, Acute respiratory distress syndrome, PI3K p110α

## Abstract

**Background:**

Telocytes (TCs) are newly identified interstitial cells that participate in tissue protection and repair. The present study investigated the mechanisms underlying the protective effect of TCs in a mouse model of respiratory distress.

**Methods:**

The mouse model of acute respiratory distress syndrome (ARDS) was established by intratracheal instillation of lipopolysaccharide (LPS). After instillation of TCs culture medium, lung injury was assessed, and angiogenesis markers, including CD31 and endothelial nitric oxide synthase (eNOS), were detected by immunofluorescence. Bioinformatics analysis was used to screen significantly differentially expressed microRNAs (miRNAs) in cultured TCs stimulated with LPS, and the regulation of downstream angiogenesis genes by these miRNAs was analysed and verified. PI3K subunits and pathways were evaluated by using a PI3K p110α inhibitor to study the involved mechanisms.

**Results:**

In ARDS mice, instillation of TCs culture medium ameliorated LPS-induced inflammation and lung injury and increased the protein levels of CD31 and eNOS in the injured lungs. A total of 7 miRNAs and 1899 mRNAs were differentially regulated in TCs stimulated with LPS. Functional prediction analysis showed that the differentially expressed mRNAs were enriched in angiogenesis-related processes, which were highly correlated with miR-21a-3p. Culture medium from TCs with miR-21a-3p inhibition failed to promote angiogenesis in mouse models of LPS-induced ARDS. In cultured TCs, LPS stimulation upregulated the expression of miR-21a-3p, which further targeted the transcription factor E2F8 and decreased Notch2 protein expression. TCs culture medium enhanced hemangioendothelioma endothelial cells (EOMA cells) proliferation, which was blocked by the miR-21a-3p inhibitor. The PI3K p110α inhibitor decreased vascular endothelial growth factor levels in LPS-stimulated TCs and reversed the enhancing effect of TCs culture medium on EOMA cells proliferation.

**Conclusions:**

TCs exerted protective effects under inflammatory conditions by promoting angiogenesis via miR-21a-3p. The PI3K p110α subunit and transcriptional factor E2F8 could be involved in this process.

## Introduction

Acute respiratory distress syndrome (ARDS) is a clinical syndrome characterised by acute progression of respiratory failure. According to an international multi-centre research, the prevalence of ARDS was 10.4% of ICU admissions [1]. Inflammatory responses destroy underlying vascular endothelial cells and respiratory epithelial cells and impair the lungs’ ability to exchange oxygen and carbon dioxide [2]. Therefore, decreasing inflammation and accelerating blood vessel repair are two key factors in the prevention and treatment of ARDS. Since its severity and lack of effective pharmacologic treatments [3], it is of great significance to explore novel therapeutic strategies for ARDS. Recently, cell therapy have been shown to have promising therapeutic potential. Mesenchymal stem cells ameliorated ARDS due to paracrine mechanism [4].

Telocytes (TCs) are newly identified mesenchymal cells that play a role in providing nutrition to surrounding cells by cell–cell communication and have post-injury repair and regeneration functions [5–7]. TCs contribute to angiogenesis within the myocardium [8]. Transplantation of cardiac TCs promotes post ischaemic myocardial repair [9]. Pulmonary TCs also assist with angiogenesis since they participate in forming the structure of the air–blood barrier [10]. Intratracheal administration of activated TCs has been reported to alleviate ventilator-induced lung injury in a mouse model by releasing angiogenic factors [11]. However, the underlying mechanism remains unclear.

Class I Phosphoinositide-3-kinases (PI3Ks) or the four subtypes of catalytic subunit—p110α, p110β, p110γ and p110δ—are expressed in all mammalian cells. The catalytic subunits bind to p85 regulatory subunits, activate receptor tyrosine kinases (RTKs), and transmit a variety of cell surface receptor signals, such as those from the epidermal growth factor receptor (EGFR) or fibroblast growth factor receptor (FGFR), to promote cell growth [12]. The PI3K subunits p110α and p110δ were demonstrated to be associated with tissue repair; however, this function is mediated by different mechanisms. The activity of PI3K p110α can be enhanced by tyrosine kinase ligands, such as vascular endothelial growth factor (VEGF) A, and can induce angiogenesis and vascular remodelling [13]. Moreover, p110α regulates endothelial cell migration through the small GTPase RhoA, mediated by *PI3KCG*, a gene encoding a p110γ subunit, which has a protective effect on hypoxic-reoxygenated cardiomyocytes mediated by activation of the PI3K/AKT signalling pathway and inhibition of apoptosis [14]. PI3K (p110δ)/AKT/mammalian target of rapamycin (mTOR) signalling pathway mediates interferon-γ (IFN-γ) induced airway epithelial cell growth and proliferation through interaction with CEACAM1 [15].

MicroRNAs (miRNAs) are small, non-coding RNAs that regulate the expression of target genes via posttranscriptional degradation of mRNA and/or translational inhibition of protein expression. MiR-135a can influence cell proliferation, migration, invasion, apoptosis and tumour angiogenesis through the IGF-1/PI3K/AKT signalling pathway in non-small cell lung cancer (NSCLC) [16]. Mature miR-21a-5p was found to be secreted by lipopolysaccharide (LPS)-activated macrophages in small vesicles, which were endocytosed and internalised by renal fibroblasts, thereby promoting the expression of fibrosis and inflammation markers in a mouse model of chronic renal allograft dysfunction (CAD) in allogeneic kidney transplantation [17]. Antagonism of miR-21a-5p ameliorated CAD in mouse model following kidney transplantation [17]. In patients with renal allograft, elevation of urinary [18] and plasma [19] miR-21 level was correlated with interstitial fibrosis and tubular atrophy.

The TCs line was established by transfection with simian vacuolating virus 40 (SV40) and identified to maintain TCs morphology and immune characteristics [20]. TCs proliferation was demonstrated to be regulated by transforming growth factor-β (TGF-β) and mediated by the PI3K p110α subunit and the PI3K/AKT/mTOR signalling pathway [21]. The present study was designed to investigate the underlying protective effect of TCs in a mouse model of respiratory distress. Bioinformatics approaches were applied to analyse gene expression profiles in TCs challenged with LPS. Particular attention was devoted to the angiogenesis-related process. The protective mechanisms mediated by the PI3K subunit in TCs were further examined in hemangioendothelioma endothelial cells (EOMA cells) in vitro. The current study presents the theoretical bases of an alternative new potential therapeutic strategy for ARDS.

## Methods

### Animal models

Eight-week-old male C57BL/6 mice, 22 to 25 g, were purchased from Shanghai Jiesijie Company (Shanghai, China). Mice were randomly divided into four groups: Control, ARDS, ARDS with negative control (NC) TCs treatment, and ARDS with miR-21a-3p inhibited TCs treatment. Under anaesthesia (60 mg/kg sodium pentobarbital, Sinopharm Chemical Reagent Co. Shanghai, China), mice were intratracheally instilled with phosphate-buffered saline or LPS (5 mg/kg, Sigma, Germany) via 20-gauge catheters. Mice in the ARDS treatment groups were also instilled with 20 μL of TCs culture medium from TCs treated with the NC or miR-21a-3p inhibitor in the presence of LPS. Twenty-four hours later, animals were sacrificed, and the lungs were collected.

The study protocol was approved by the Animal Ethics Committee of Zhongshan Hospital, Fudan University.

### TCs

Mouse primary pulmonary TCs were a kind gift from Dr. Dongli Song. TCs were cultured in Dulbecco’s modified Eagle’s medium/F12 (DMEM/F12, Hyclone, Boston, MA) supplemented with 5% foetal bovine serum (FBS, Cellsera, Australia). Experiments with LPS (0.1 μg/mL) were performed in DMEM/F12 without FBS. TCs culture medium was collected from culture dishes after LPS stimulation for 48 h. The p110α inhibitor HS-173 (Selleck, Shanghai, China) was applied 2 h before LPS stimulation.

### MiRNA transfection

Both the miR-21a-3p inhibitor and NC were purchased from China Ribobio (Ribobio, Guangzhou, China). TCs were transfected with the miR-21a-3p inhibitor and NC at a final concentration of 50 nmol/L using a lipofectamine RNAiMAX transfection system (ThermoFisher Scientific, Carlsbad, CA) according to the manufacturer’s protocol. Cells were incubated with siRNA in serum-free and antibiotic-free medium for 6 h and then in normal growth medium for another 24 h before the experiments were performed.

### Gene expression profiling analysis

Gene expression profiling analysis of both miRNA and mRNA were performed with Agilent Microarray Scanner (Cat # G2565CA, Agilent technologies, Santa Clara, CA). The data were normalised with the *AgiMicroRna* package [22]. The gene expression files were analysed with R-3.4.1 software. Differentially expressed genes (DEGs) were defined as those with an adjusted P-value of less than 0.05. DEGs were further analysed with the *limma* package [23]. Heat maps were generated with the *ggplot2* package [24].

The online databases miRWalk [25] and TargetScan [26] were used to screen potential miRNA target genes. Overlapping genes in the two databases were selected for further analysis. The online database STRING [27] and the Database for Annotation, Visualization and Integrated Discovery (DAVID) v6.8 [28] were used to analyse gene function. The relationship between DEGs and miRNAs was further visualised with Cytoscape 3.7.1 [29].

### Quantification of mRNA and miRNA

Total RNA was extracted from cultured TCs with TRIzol (Takara, Shiga, Japan) according to the provided instructions. MiRNAs were reverse transcribed with a Bulge-Loop miRNA qRT-PCR Starter Kit (Ribobio, Guangzhou, China), and mRNAs were reverse transcribed to complementary DNA (cDNA) with a PrimeScript RT Reagent Kit with gDNA Eraser (Takara, Shiga, Japan). The expression levels of miR-21a-3p, miR-221-5p and mRNAs were measured by quantitative real-time polymerase chain reaction (qPCR) on a Bio-Rad IQ5 real-time PCR instrument, with U6 and GAPDH used as the housekeeping genes for miRNAs and mRNAs, respectively. MiRNA PCR was performed with the Bulge-Loop miRNA qRT-PCR Starter Kit, Bulge-Loop mmu-miR-21a-3p Primer Set and Bulge-Loop mmu-miR-221-5p Primer Set (Ribobio, Guangzhou, China). MRNA primers were synthesised by Sangon (Shanghai, China). The following mouse-specific primers were used: GAPDH sense primer: 5′-GTTCAACGGCACAGTCAAG-3′, antisense primer: 5′-GCCAGTAGACTCCACGACAT-3′; E2F8 sense primer: 5′-CTGTTT GCACGAACACTTATCAG-3′, antisense primer: 5′-GTACCGCGCTAGGAATTTGTG-3′; Acvrl1 sense primer: 5′-TGATTCCTGTTGCCGGCCT-3′, antisense primer: 5′-CAGTGTGGGCTCTCACAAGT-3′; Rbpj sense primer: 5′-TGGCGAGAGTTTGTGGAAGA-3′, antisense primer: 5′-AGCACTGTTTGATCCCCTCG-3′; Notch1 sense primer: 5′-TGTGGCTTCCTTCTACTGCG-3′, antisense primer: 5′-CTTTGCCGTTGACAGGGTTG-3′; Flt1 sense primer: 5′-GTGAGCACTGCGGCAAAAAG-3′, antisense primer: 5′-ACTCATTTTGGGAGGAGCGT -3′; EFNB2 sense primer: 5′-CGAGGTGGCAACAACAATGG-3′, antisense primer: 5′-ATAGTCCCCGCTGACCTTCT -3′; Thbs1 sense primer: 5′-CTGCCAATCATAACCAGCG-3′, antisense primer: 5′-TTCGTTAAAGGCCGAGTGCT-3′; EPAS1 sense primer: 5′-CTGAGGAAGGAGAAATCCCGT-3′, antisense primer: 5′-TGTGTCCGAAGGAAGCTGATG-3′; hypoxia inducible factor-1α (HIF-1α) sense primer: 5′-ACCTTCATCGGAAACTCCAAAG-3′, antisense primer: 5′-CTGTTGGCTGGGAAAAGTTAGG-3′; PIK3CA sense primer: 5′-CCACGACCATCTTCGGGTG-3′, antisense primer: 5′-ACGGAGGCATTCTAAAGTCACTA-3′; PIK3CB sense primer: 5′-CTATGGCAGACAACCTTGACAT-3′, antisense primer: 5′-CTTCCCGAGGTACTTCCAACT-3′; PIK3CD sense primer: 5′-GTAAACGACTTCCGCACTAAGA-3′, antisense primer: 5′-GCTGACACGCAATAAGCCG-3′; and VEGF sense primer: 5′-GTACCTCCACCATGCCAAGT-3′, antisense primer: 5′-TCCTATGTGCTGGCTTTGGT-3′.

### Western blotting

Total protein was extracted from cultured TCs with lysis buffer (150 mmol/L NaCl, 1 mmol/L EDTA, 1 mmol/L NaF, 1 mmol/L dithiothreitol, 10 μg/μL aprotinin, 10 μg/μL leupeptin, 0.1 mmol/L Na3VO4, 1 mmol/L phenylmethylsulfonyl fluoride (PMSF), and 0.5% NP-40). Protein extracts (20 μg) were separated by 10% sodium dodecyl sulfate-polyacrylamide gel electrophoresis and transferred to polyvinylidene fluoride membranes (Merck Millipore, Darmstadt, Germany). After blocking with 5% non-fat milk/Tris-buffered saline containing 0.1% Tween 20 at room temperature for one hour, membranes were incubated with primary antibodies [specific for GAPDH (60004-1-Ig, Proteintech, Wuhan, China), E2F8 (ab109596, Abcam, Cambridge, UK), Delta-like 4 (DLL4)(ab7280, Abcam, Cambridge, UK), Notch1 (sc-373891, Santa Cruz, Dallas, TX), Notch2 (sc-5545, Santa Cruz, Dallas, TX), Notch4 (sc-5594, Santa Cruz, Dallas, TX), phosphatase and tensin homolog deleted on chromosome ten (PTEN)(ab32199, Abcam, Cambridge, UK), PI3K (4257T, CST, Boston, MA), p-PI3K (4228T, CST, Boston, MA), mTOR (2983T, CST, Boston, MA), p-mTOR (5536T, CST, Boston, MA), AKT (9272S, CST, Boston, MA), p-AKT (9271S, CST, Boston, MA), and p110α (4249T, CST, Boston, MA)] overnight at 4 °C. Protein expression levels were normalised to those of GAPDH with ImageJ (NIH, Bethesda, MD).

### EOMA cells proliferation assay

EOMA cells proliferation was assessed with a colorimetric assay—Cell Counting Kit-8 (CCK8, Yeasen, Shanghai, China)—following the manufacturer’s protocol. Approximately 4000 EOMA cells/well were seeded in a 96-well plate. After adhesion, EOMA cells were incubated for 24 h with culture medium from TCs transfected with the miR-21a-3p inhibitor or NC in the presence of LPS.

### Dual luciferase assay

The pGL3 reporter vector (Promega, Madison, WI) was used to generate the plasmids pGL3-WT-E2F8-3′-UTR and pGL3-Mut-E2F8-3′-UTR. Human embryonic kidney cells were co-transfected with pGL3-E2F8-3′-UTR (WT or Mut) and the miR-21a-3p mimic or NC with Lipofectamine 2000 reagent (ThermoFisher Scientific, Carlsbad, CA). After incubation for 24 h, luciferase activity was assessed by the Dual-Luciferase Reporter Assay System (Promega, Madison, WI) according to the manufacturer’s protocol.

### Enzyme-linked immunosorbent assay (ELISA)

The concentration of VEGF in the TCs culture medium was measured by a commercial VEGF ELISA kit (Westang, Shanghai, China) according to the manufacturer’s protocol.

### Dynamic real-time cell observation

Live observation of EOMA cells was performed with a Cell-IQ cell culture platform (Chip-Man Technologies, Tampere, Finland) equipped with a phase contrast microscope (Nikon CFI Achromat phase contrast objective with 10 magnification) and a camera (Nikon, Fukasawa, Japan). The equipment was controlled by Imagen software (Chip-Man Technologies). Each group contained 16 replicates of visual fields. Images were acquired at 1-h intervals for 48 h.

### Tissue preparation and immunofluorescence examination

Lung tissues were fixed with 10% formalin solution and embedded in paraffin. Each tissue was sectioned at 5 μm and stained with haematoxylin–eosin (HE, Beyotime, Shanghai, China) according to the manufacturer’s protocol. For immunofluorescence staining, an antigen retrieval protocol was carried out with incubation in 0.3% H_2_O_2_ for 30 min and heating to boiling in a microwave in citrate buffer for 10 min. After blocking with 5% goat serum in Tris-buffered saline, sections were incubated with diluted primary antibodies [CD31 (1:500, ab24590, Abcam, Cambridge, UK), endothelial nitric oxide synthase (eNOS) (1:500, Cat610296, BD Biotechnology, San Jose, CA)] overnight at 4 °C and then with secondary antibodies and 4′,6-diamidino-2-phenylindole (DAPI), separately.

### Statistical analysis

Data are expressed as the means ± SDs and were analysed by one-way analysis of variance (ANOVA) and Tukey’s multiple comparisons test. A P-value of < 0.05 was considered statistically significant. All statistical analyses were performed with GraphPad Prism 7.04 (GraphPad, San Diego, CA).

## Results

### Protective effects of TCs in ARDS

**Fig. 1:**
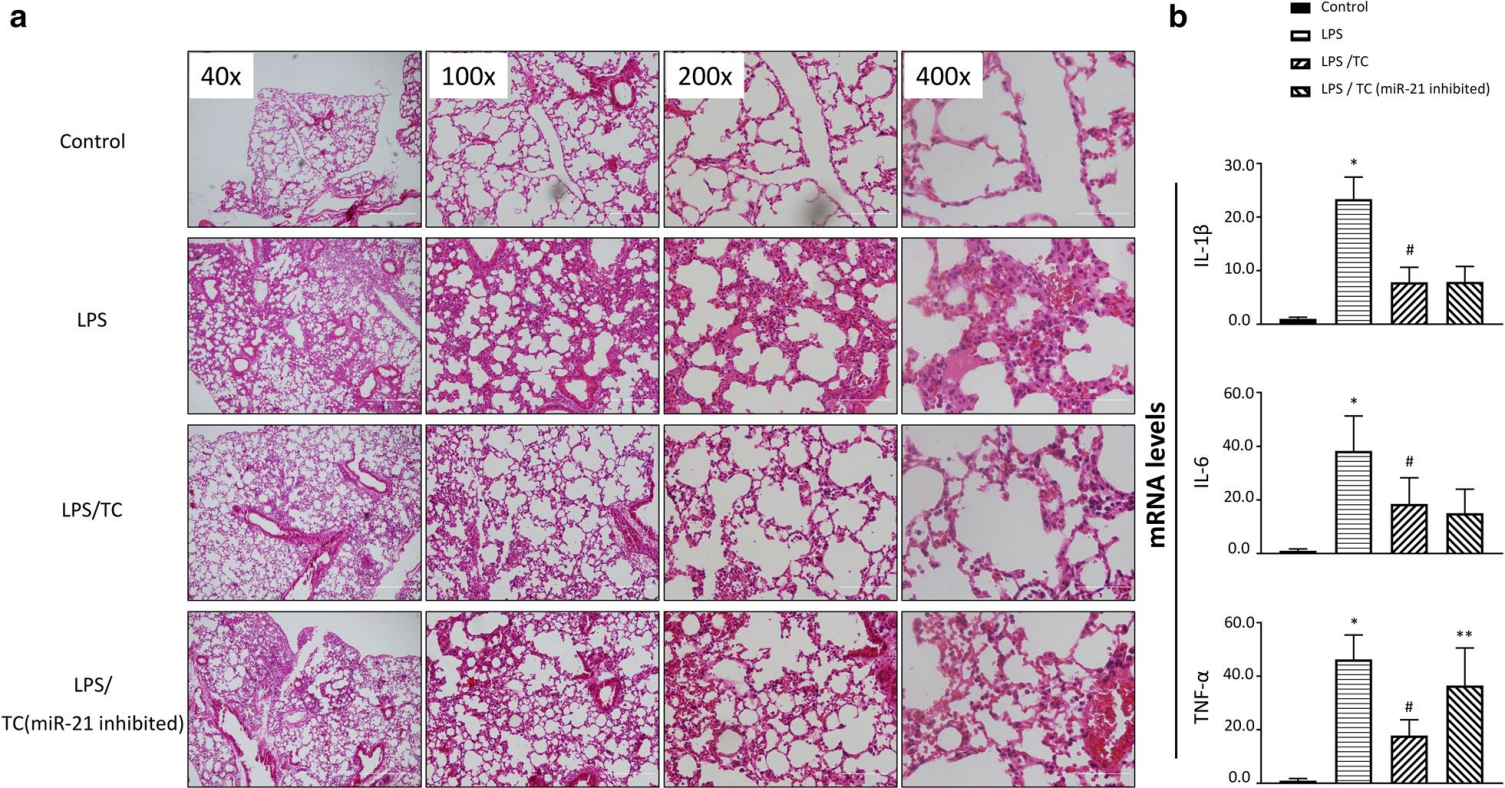
Protective effects of TCs in ARDS mouse models. **a** HE staining of mice lung tissue in lungs of control mice (Control), ARDS mice (LPS), ARDS mice treated with TCs supernatant (LPS/TC), and ARDS mice treated with miR-21a-3p inhibited TCs supernatant [LPS/TC (miR-21 inhibited)]. **b** The mRNA expression of inflammatory cytokines in mice lungs in the above four groups. *P < 0.05 vs Control, ^*#*^*P *< 0.05 vs LPS, **P < 0.05 vs LPS/TCs, n = 6. *IL-1β* interleukin-1β, *IL-6* interleukin-6, *TNF-α* tumour necrosis factor-α

The ability for TCs protection was first estimated in ARDS mouse models. LPS stimulation caused inflammatory infiltration, alveolar wall widening, and vessel destruction (Fig. 1a). The production of inflammatory cytokines was elevated in ARDS mice (Fig. 1b). Since substances, including molecules and exosomes, released by TCs could be important factors affecting adjacent cells, the effect of TCs culture medium was assessed. Instillation of TCs culture medium reduced the inflammatory infiltration, reduced the alveolar interstitial width and decreased the levels of inflammatory cytokines. Bio-behaviours of TCs were recorded by Cell-IQ to show the typical morphology of cultured cells (Additional file 1: Figure S1).

### TCs promoted angiogenesis in ARDS

**Fig. 2:**
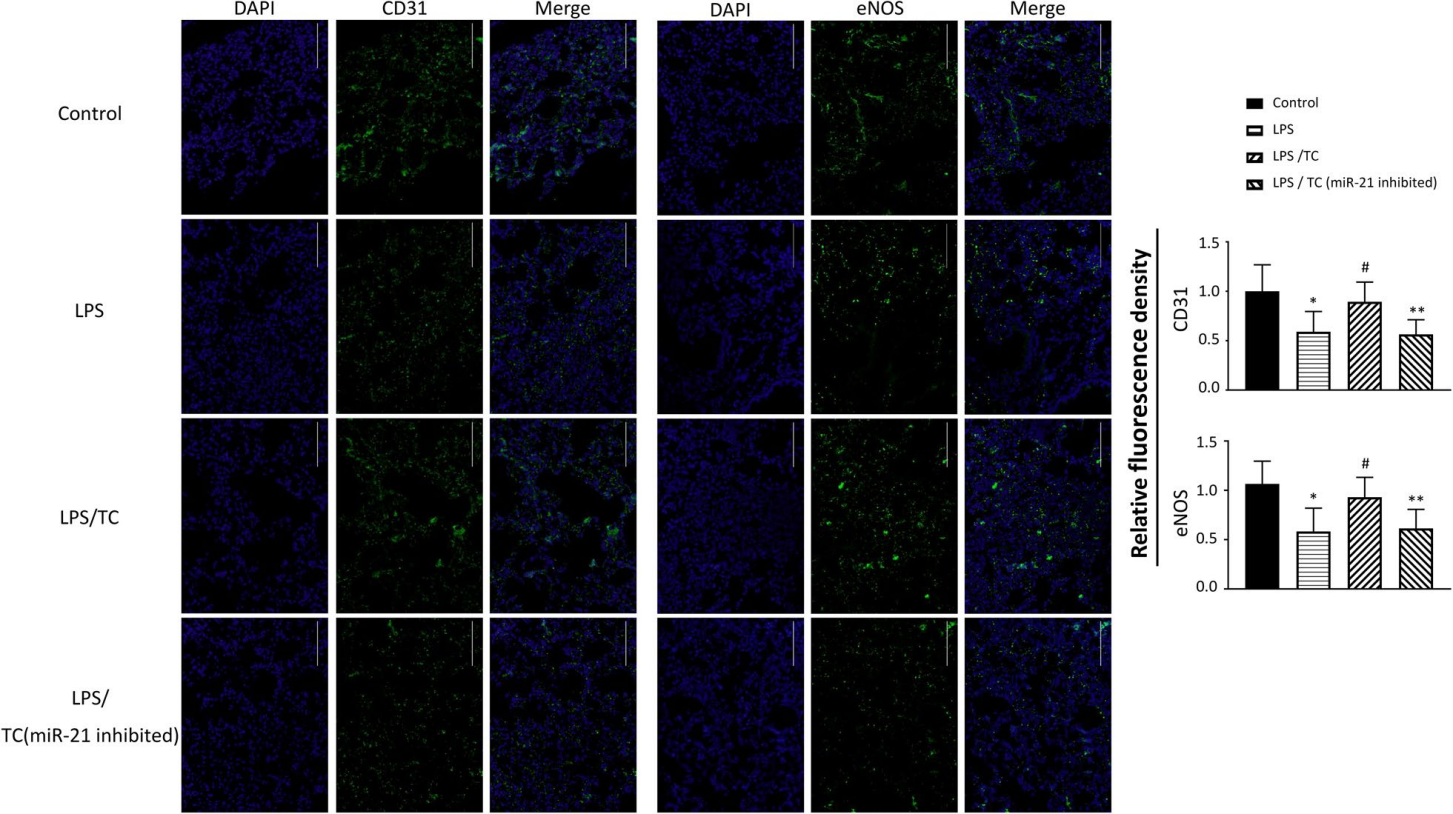
TCs promoted angiogenesis in ARDS mouse models. The expression of CD31 and eNOS in lungs of control mice (Control), ARDS mice (LPS), ARDS mice treated with TCs supernatant (LPS/TC), and ARDS mice treated with miR-21a-3p inhibited TCs supernatant [LPS/TC (miR-21 inhibited)] was shown in green fluorescence, dapi was blue. *P < 0.05 vs Control, ^*#*^*P *< 0.05 vs LPS, **P < 0.05 vs LPS/TCs, n = 6. Original magnification: 200x

As angiogenesis is essential in tissue repair, the induction of angiogenic factors in TCs after stimulation with LPS was assessed. In ARDS mice, the expression of the angiogenesis-related marker CD31 and eNOS was downregulated. However, an increase in CD31 and eNOS expression was observed in the WT TCs treatment group but not in the group treated with medium from TCs with miR-21a-3p inhibition (Fig. 2).

### MiRNA and mRNA profiles in LPS-stimulated TCs

**Fig. 3:**
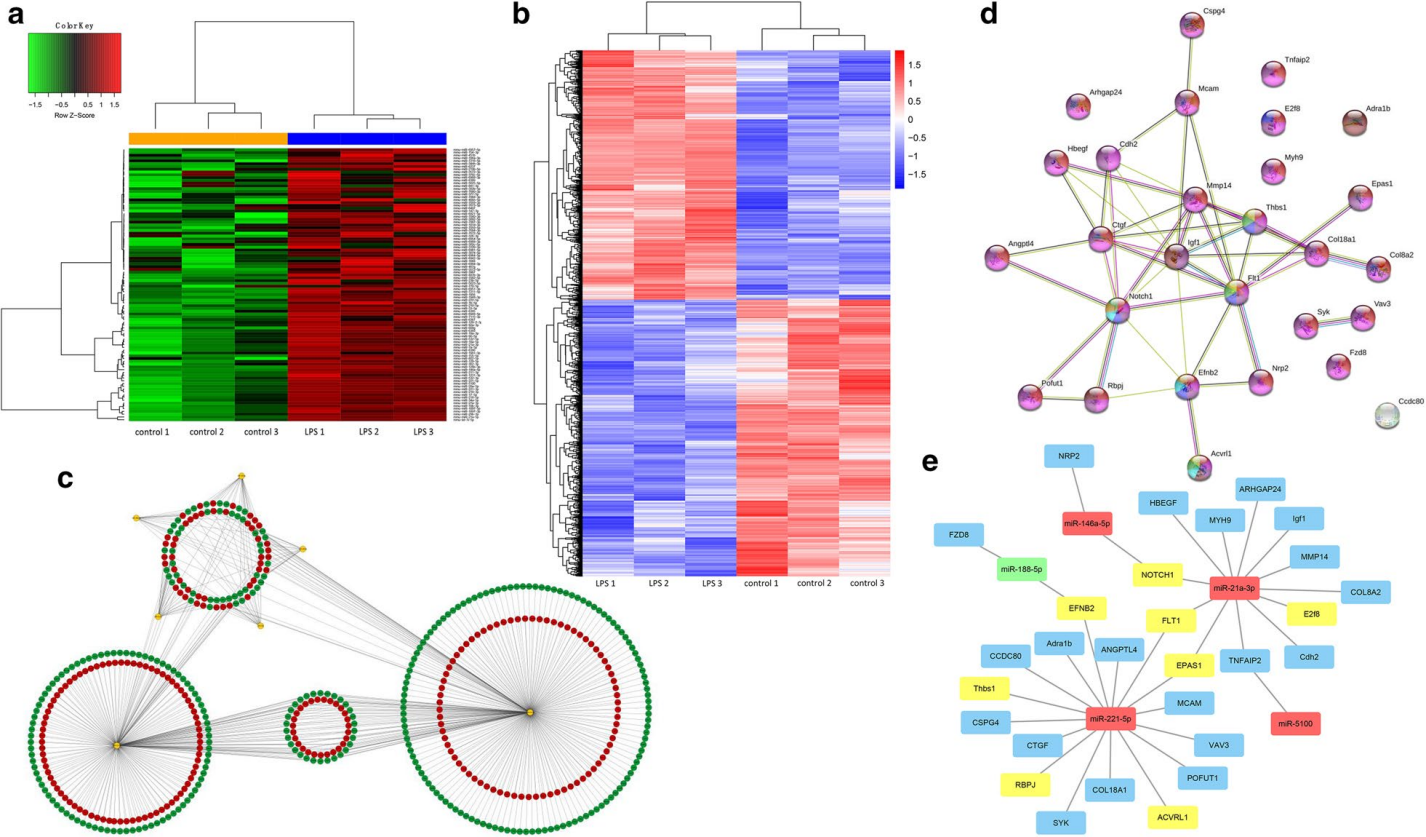
Differentially expressed genes in TCs with LPS treatment. **a** Heat map of differential expressed miRNAs in cultured TCs stimulated with LPS. **b** Heat map of differential expressed genes in cultured TCs stimulated with LPS. **c** Relationship between differential expressed miRNAs and their differential expressed target mRNAs. Yellow indicated miRNAs, green indicated downregulated mRNAs, and red indicated upregulated mRNAs. **d** Interaction of the angiogenesis-related proteins in STRING. Different colours indicated the involvement of proteins in different processes. Red indicated angiogenesis, pink indicated blood vessel morphogenesis, purple indicated vasculature development, brown indicated blood vessel remodelling, blue indicated sprouting angiogenesis, cyan indicated venous blood vessel sprouting, orange indicated venous blood vessel morphogenesis, green indicated regulation of angiogenesis, and yellow indicated positive regulation of angiogenesis. **e** Angiogenesis related miRNAs and their downstream genes. Red indicated upregulated miRNAs, green indicated downregulated miRNAs, yellow indicated genes that were enriched in more than three processes in STRING, blue indicated other mRNAs

To identify the critical miRNAs in the regulation of angiogenesis by TCs, miRNA and mRNA profiles were generated, and the relationship of differentially expressed miRNAs with downstream angiogenesis factor-associated mRNAs were analysed in LPS-stimulated TCs. In LPS-stimulated TCs, six miRNAs, including miR-155-5p, miR-21a-3p, miR-5100, miR-221-5p, miR-7a-3p and miR-146a-5p, were upregulated, and one miRNA (miR-188-5p) was downregulated with an absolute fold change > 2 (Fig. 3a and Table 1). By referring these results to two online databases (miRWalk and TargetScan), 4368 target genes were predicted to be downstream targets of the differentially expressed miRNAs.

**Table 1:** Summary of differentially expressed miRNAs in TCs treated with LPS
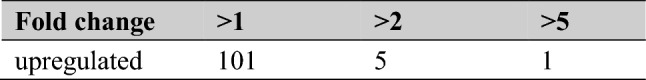

Gene symbols	Fold change	Gene symbols	Fold change	Gene symbols	Fold change
mmu-miR-146a-5p	8.761	mmu-miR-1231-3p	1.378	mmu-miR-6237	1.067
mmu-miR-7a-5p	2.813	mmu-miR-1948-3p	1.370	mmu-miR-7059-3p	1.065
mmu-miR-221-5p	2.407	mmu-let-7i-5p	1.350	mmu-miR-881-3p	1.064
mmu-miR-5100	2.330	mmu-miR-29b-3p	1.347	mmu-miR-3074-5p	1.063
mmu-miR-21a-3p	2.258	mmu-miR-3967	1.337	mmu-miR-7216-5p	1.063
mmu-miR-155-5p	2.067	mmu-miR-487b-3p	1.333	mmu-miR-770-5p	1.063
mmu-miR-33-5p	1.947	mmu-miR-1981-3p	1.300	mmu-miR-6901-5p	1.062
mmu-miR-96-5p	1.930	mmu-miR-6948-5p	1.299	mmu-miR-3092-5p	1.062
mmu-miR-532-3p	1.901	mmu-miR-6395	1.292	mmu-miR-302c-5p	1.061
mmu-miR-129-5p	1.845	mmu-miR-21a-5p	1.287	mmu-miR-6988-3p	1.060
mmu-miR-532-5p	1.837	mmu-miR-6367	1.263	mmu-miR-3109-3p	1.058
mmu-miR-211-3p	1.771	mmu-miR-7002-5p	1.251	mmu-miR-1668	1.057
mmu-miR-19a-3p	1.768	mmu-miR-27a-3p	1.219	mmu-miR-3572-3p	1.055
mmu-miR-6951-3p	1.766	mmu-miR-5623-5p	1.203	mmu-miR-451b	1.054
mmu-miR-210-3p	1.763	mmu-miR-23b-5p	1.190	mmu-miR-7007-3p	1.051
mmu-miR-126b-3p	1.756	mmu-miR-302b-5p	1.186	mmu-miR-6957-5p	1.048
mmu-miR-221-3p	1.738	mmu-miR-669g	1.181	mmu-miR-219b-5p	1.045
mmu-miR-6369	1.713	mmu-miR-1b-5p	1.167	mmu-miR-377-5p	1.043
mmu-miR-92a-3p	1.712	mmu-miR-147-3p	1.158	mmu-miR-6921-5p	1.043
mmu-miR-18a-5p	1.675	mmu-miR-6994-3p	1.123	mmu-miR-7040-3p	1.043
mmu-miR-802-5p	1.662	mmu-miR-7680-3p	1.114	mmu-miR-7068-3p	1.042
mmu-miR-1897-5p	1.631	mmu-miR-7673-5p	1.101	mmu-miR-376c-5p	1.040
mmu-miR-129-2-3p	1.615	mmu-miR-6908-3p	1.090	mmu-miR-7059-5p	1.039
mmu-miR-7211-5p	1.611	mmu-miR-3572-5p	1.088	mmu-miR-7684-3p	1.038
mmu-miR-210-5p	1.594	mmu-miR-106a-3p	1.084	mmu-miR-181d-3p	1.037
mmu-miR-362-3p	1.576	mmu-miR-6964-5p	1.083	mmu-miR-6942-3p	1.036
mmu-miR-218-5p	1.540	mmu-miR-6407	1.082	mmu-miR-511-3p	1.034
mmu-miR-6396	1.537	mmu-miR-6954-5p	1.081	mmu-miR-741-3p	1.034
mmu-miR-17-5p	1.517	mmu-miR-344h-3p	1.081	mmu-miR-465d-5p	1.029
mmu-miR-1897-3p	1.512	mmu-miR-467g	1.080	mmu-miR-1955-3p	1.029
mmu-miR-1956	1.479	mmu-miR-154-3p	1.080	mmu-miR-6930-5p	1.027
mmu-miR-20a-5p	1.475	mmu-miR-5625-5p	1.080	mmu-miR-7013-5p	1.025
mmu-miR-34a-5p	1.413	mmu-miR-6389	1.076	mmu-miR-3109-5p	1.025
mmu-miR-222-5p	1.401	mmu-miR-466n-5p	1.071	mmu-miR-7089-3p	1.022
mmu-miR-19b-3p	1.390	mmu-miR-326-3p	1.071	mmu-miR-375-5p	1.018
mmu-miR-7115-3p	1.378	mmu-miR-7672-5p	1.069		


Gene symbol	Fold change	Gene symbol	Fold change	Gene symbol	Fold change
mmu-miR-188-5p	0.393	mmu-miR-6922-3p	0.734	mmu-miR-139-3p	0.908
mmu-miR-291a-3p	0.503	mmu-miR-202-3p	0.734	mmu-miR-184-3p	0.910
mmu-miR-375-3p	0.520	mmu-miR-6936-5p	0.735	mmu-miR-381-3p	0.911
mmu-miR-6974-3p	0.527	mmu-miR-6960-3p	0.737	mmu-miR-3471	0.911
mmu-miR-5107-5p	0.528	mmu-miR-125a-3p	0.739	mmu-miR-299b-5p	0.916
mmu-miR-7020-5p	0.544	mmu-miR-7005-5p	0.749	mmu-miR-6546-3p	0.919
mmu-miR-6948-3p	0.569	mmu-miR-691	0.759	mmu-miR-5116	0.930
mmu-miR-7014-5p	0.572	mmu-miR-6364	0.769	mmu-miR-376a-3p	0.937
mmu-miR-328-5p	0.576	mmu-miR-6929-3p	0.780	mmu-miR-292a-3p	0.938
mmu-miR-434-3p	0.576	mmu-miR-5622-3p	0.788	mmu-miR-1943-3p	0.939
mmu-miR-466h-3p	0.581	mmu-miR-1192	0.790	mmu-miR-7078-5p	0.939
mmu-miR-877-5p	0.591	mmu-miR-5627-5p	0.792	mmu-miR-7024-3p	0.943
mmu-miR-6981-3p	0.595	mmu-let-7j	0.797	mmu-miR-6398	0.944
mmu-miR-1907	0.606	mmu-miR-5624-3p	0.819	mmu-miR-466m-5p	0.946
mmu-miR-6976-5p	0.625	mmu-miR-5107-3p	0.822	mmu-miR-6984-5p	0.946
mmu-miR-3087-3p	0.626	mmu-miR-574-5p	0.825	mmu-miR-7228-5p	0.950
mmu-miR-7019-5p	0.635	mmu-miR-3057-5p	0.842	mmu-miR-323-3p	0.951
mmu-miR-7055-3p	0.645	mmu-miR-7220-3p	0.843	mmu-miR-6394	0.957
mmu-miR-1258-5p	0.659	mmu-miR-6929-5p	0.857	mmu-miR-92a-2-5p	0.958
mmu-miR-1193-3p	0.672	mmu-miR-5124b	0.858	mmu-miR-1946a	0.962
mmu-miR-129b-5p	0.690	mmu-miR-7066-5p	0.858	mmu-miR-5620-3p	0.967
mmu-miR-345-3p	0.691	mmu-miR-335-5p	0.859	mmu-miR-18a-3p	0.969
mmu-miR-344d-3p	0.713	mmu-miR-680	0.861	mmu-miR-7676-3p	0.971
mmu-miR-693-3p	0.729	mmu-miR-325-3p	0.886	mmu-miR-346-5p	0.972
mmu-miR-678	0.730	mmu-miR-7023-3p	0.889	mmu-miR-6346	0.972
mmu-miR-7034-3p	0.730	mmu-miR-467c-3p	0.889	mmu-miR-30b-3p	0.984
mmu-miR-466l-3p	0.731				

In total, 1899 mRNAs—901 upregulated and 998 downregulated—were differentially expressed in TCs after LPS stimulation (Fig. 3b and Table 2). A total of 519 genes overlapped with those from the online prediction (Fig. 3c).

**Table 2:** Summary of differentially expressed mRNAs in TCs treated with LPS
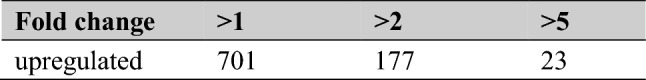

Gene symbol	Fold change	Gene symbol	Fold change	Gene symbol	Fold change
Saa3	88.57679	Sh3kbp1	1.714612	Aldoa	1.358346
Steap4	39.69958	Gm10382	1.713579	Bcl2l11	1.358341
C3	27.54466	Sphk1	1.711903	Wnt5a	1.358042
Cxcl1	21.9035	Tnfsf10	1.709923	Sdc4	1.357917
Lcn2	18.11751	Fosl2	1.70942	Pttg1	1.357846
Cp	13.17588	Arhgap24	1.703888	Eps8	1.356005
Ccl2	9.407872	Rspo3	1.702523	Parp8	1.355285
Ccl7	9.313701	Rasl11a	1.701653	AI413582	1.355264
Lbp	9.248471	Snhg11	1.699314	H2afj	1.35191
Slpi	8.496525	Pnp2	1.69892	Tapbpl	1.350347
Hp	7.020443	9930111J21Rik2	1.697292	Fam46a	1.349368
Casp4	6.491574	F730043M19Rik	1.696927	Gpr162	1.349043
Oas3	6.472296	Gstt1	1.694392	Itpr2	1.347899
Slc16a2	6.293287	Tor3a	1.694311	Fendrr	1.347418
Kng2	6.13421	Tnfrsf14	1.693478	Eif2ak2	1.346754
Neurl3	6.123261	Pcdhgc5	1.692342	Bcam	1.346359
Lgi2	5.548539	Usp18	1.690192	Tmem192	1.346301
Zbp1	5.500857	Icam1	1.685666	Atxn7l1	1.346144
Cebpd	5.490325	Vnn1	1.684905	Tcirg1	1.346103
Tmem176a	5.352542	Isg20	1.684825	H6pd	1.34602
Tmem176b	5.292897	Cdh23	1.682662	Acad10	1.345811
Cxcl5	5.105818	Hivep2	1.678786	Ggta1	1.344527
Serpina3i	5.062072	Serpinb9	1.676066	Ago4	1.343551
Ly6a	4.890107	Ier3	1.670507	Rpl39	1.343105
Ms4a4d	4.778005	Spidr	1.66764	Arsj	1.342469
Kcnj15	4.627825	Parp10	1.667283	Kif21a	1.341944
Slfn2	4.500739	Jak2	1.667282	Grtp1	1.340769
Nfkbiz	4.420487	Lnx1	1.665956	Hook2	1.340696
Oas1g	4.352056	Dtx3l	1.665121	Pisd-ps1	1.340025
Gm8995	4.258631	Hopx	1.661591	Hbp1	1.33937
Zc3h12a	4.118584	Rsl1	1.6613	Mlkl	1.338835
Mt2	4.050379	Insig2	1.660402	Fbln1	1.338393
H2-Q7	4.021978	Syt17	1.659707	Ern1	1.338122
Kank4	3.966313	Ppp1r3b	1.659039	Unc93b1	1.338107
Gm16685	3.952599	Junb	1.658968	Oplah	1.337688
Phf11b	3.899771	Lhfpl2	1.658507	Dhrs9	1.337432
H2-Q5	3.876445	Aqp3	1.658025	Ank3	1.336823
Gbp5	3.860209	Bst1	1.653379	Hspa1a	1.335089
Gbp3	3.711967	Cd14	1.647933	Fos	1.334711
Xdh	3.706347	Abcb1a	1.64782	Dusp1	1.334023
Sod3	3.705108	Gm43068	1.642165	Tmem53	1.332695
H2-T10	3.615936	Tsc22d1	1.640017	Riok3	1.332508
Adamts7	3.547664	Cfap69	1.639558	Zc2hc1a	1.332429
H2-K1	3.536976	Clca3a1	1.637745	Gata6	1.33174
Ccl5	3.523568	En1	1.637662	Nrp2	1.330772
Lrrc32	3.510921	Pdk1	1.637498	Fam134b	1.330567
Ntn1	3.468896	Pnrc1	1.636296	Dgat2	1.330413
Fas	3.466618	Enpp2	1.635831	Trafd1	1.328525
H2-Q6	3.433254	Abcd2	1.63519	Laptm4b	1.328518
Vcam1	3.350214	Gm16365	1.634922	D930015E06Rik	1.328021
Cd74	3.317107	Fabp4	1.634064	Timp1	1.327406
Ch25h	3.315495	Parp9	1.633709	Itga7	1.327246
Psmb8	3.278491	Serpinb1b	1.63368	Galk2	1.326399
Il6	3.219911	Gm13010	1.633034	Rhbdl3	1.325887
Gm4951	3.2116	Stx6	1.632089	Cdkn2b	1.324984
H2-T23	3.207359	Elf3	1.632065	Psme2	1.324769
Plac8	3.197626	Spp1	1.630536	Sh3bp5	1.32441
Oas2	3.197366	Rnf144a	1.630365	Mif	1.323852
Arrdc4	3.158372	Trp63	1.62831	Pgk1	1.323539
C1ra	3.158361	A330074K22Rik	1.626237	Fam43a	1.322902
Gbp6	3.147647	Sfmbt2	1.625612	Arid3a	1.321845
Slc11a2	3.134662	Gbp11	1.625111	Lars2	1.321181
Ppm1h	3.127213	Ifih1	1.622407	Morc3	1.320638
Ifi47	3.105228	Tnnc1	1.620872	Shb	1.319344
Ccl20	3.098563	Tmem86a	1.61825	Sat1	1.318761
Ifi205	3.098305	Gm15433	1.615772	Acvr1b	1.318652
F830016B08Rik	3.053166	Enpp4	1.615508	Tmem170b	1.317625
Oas1a	3.019199	Trim5	1.614655	Hadh	1.317598
Ifi203	3.016137	Serpinb9b	1.608848	Stat5a	1.315396
Map3k8	3.013409	C130074G19Rik	1.606202	2-Mar	1.314095
Gm12250	3.012671	AI854703	1.605264	Eno2	1.313938
Tgtp2	3.005529	Tuba8	1.604317	Enpp5	1.313706
H2-Q4	2.972546	Serping1	1.603547	Irak4	1.312126
Mx1	2.865782	Il13ra1	1.602487	Rsrp1	1.31211
Uba7	2.864864	Piwil4	1.602483	Kcnab2	1.311973
Mmp19	2.861314	Rhbdl2	1.601725	Ptgr1	1.311529
Psmb9	2.850848	Fst	1.600533	Elf1	1.311246
Slc7a2	2.846346	Trim34a	1.600318	Ablim1	1.311022
Tspan11	2.844123	Amigo2	1.599439	Tnfaip6	1.310247
Tnn	2.822897	Hcn1	1.597937	Socs2	1.309989
H2-D1	2.809787	Egfr	1.592054	Pisd-ps2	1.308864
AI607873	2.803398	Hpse	1.588624	Traf2	1.308431
Rsad2	2.794983	AW011738	1.587681	Tfrc	1.308415
C1s1	2.784179	Dpep1	1.587215	Nadk	1.308229
Nod2	2.775693	Pydc3	1.583759	Acacb	1.306549
Sod2	2.762665	Tnfaip2	1.583296	Fbxl5	1.305723
Apol6	2.760061	Irgm1	1.583039	Slc2a1	1.304708
Ifi44	2.759968	Rnd1	1.582149	Zeb2	1.304438
Nfkbia	2.75616	Aldoc	1.581229	Ada	1.302207
Irf7	2.751557	Lrp1	1.579985	Rpl38	1.30147
Bmp3	2.745035	Ninl	1.579937	Plod2	1.30112
Kng1	2.741277	Mgarp	1.579404	Itm2c	1.300462
Cxcl10	2.723592	Gm26669	1.577867	Galnt18	1.300344
Olfr56	2.707222	Rasl11b	1.577414	Cdkn2a	1.300124
Sp100	2.654866	N4bp2l1	1.577031	Jade2	1.299487
Scube1	2.653081	Ikbke	1.573942	Cd320	1.297584
Ak4	2.651385	E230016K23Rik	1.573215	A430105I19Rik	1.296067
B2m	2.641611	Nsun7	1.57274	Cir1	1.295174
Bcl3	2.637123	Fam162a	1.568999	Rnaset2b	1.295007
Gch1	2.620331	Col18a1	1.566809	Pnpt1	1.29442
Angpt1	2.617582	Oas1b	1.563128	Eif3e	1.293846
Pdzrn4	2.608996	Bid	1.561949	Lamp2	1.292359
Ifit3	2.605192	Lipa	1.559201	Itm2b	1.291908
Serpina3h	2.599538	Dock10	1.558234	Enah	1.291837
Dram1	2.576206	Tnfsf13b	1.556325	Pou6f1	1.289991
D030025P21Rik	2.575076	Smim4	1.555893	Fibin	1.289861
Trim30a	2.570312	Gdap10	1.555756	Rgs3	1.289133
Gm5345	2.547477	Gm16217	1.554982	Btg2	1.289051
Phf11d	2.537169	Gng12	1.554866	Naa25	1.288593
Rac3	2.52761	Ddx58	1.554735	Notch3	1.286227
Cxcl3	2.52009	Fam129c	1.553522	Pcmtd2	1.28613
Pik3r5	2.519794	Dhx58os	1.550817	Tacc1	1.284319
Klf15	2.498912	Tsku	1.546568	Arfgef2	1.283314
Gbp9	2.49656	Heatr9	1.541554	Nqo2	1.283077
Wisp2	2.49624	Il6st	1.540291	Dnajb6	1.282975
Angptl4	2.493561	Stat1	1.538849	Ksr1	1.282116
Parp14	2.487984	Stap2	1.537892	Rictor	1.281955
Npy1r	2.487878	Tnip1	1.536153	Azi2	1.281312
Ecscr	2.486491	Junos	1.535682	Narf	1.280799
Tcp11l2	2.482509	Gm43050	1.534956	Aebp1	1.280756
Bst2	2.478323	Parp12	1.534759	Scarb2	1.279926
Lrig1	2.464502	Medag	1.534548	Rras	1.279648
Repin1	2.457953	Ifnlr1	1.534026	Zfp322a	1.279547
Mgst1	2.451896	Il18	1.533373	Renbp	1.279486
Ltbp2	2.441692	Adar	1.532874	Zfp263	1.278938
Fmo1	2.437275	Shisa5	1.532173	Cd302	1.278793
Mndal	2.437132	Rarres2	1.530833	Uaca	1.2784
Ifitm3	2.433955	Mitf	1.530693	Plgrkt	1.278267
Serpinb1a	2.429138	Hif1a	1.52939	Ptges	1.278224
Lgals3bp	2.420976	Znfx1	1.528807	Ezh1	1.277255
Ifi204	2.394185	Pik3r1	1.52783	Ifnar1	1.275384
Gbp2	2.374173	Grem1	1.526552	Slc25a37	1.275033
Ddx60	2.365483	Gm12216	1.520431	Arel1	1.274603
Ifit3b	2.349228	Igfbp7	1.519077	Zfp36	1.274392
Gm4070	2.333254	AI429214	1.517825	Rab11fip1	1.273259
Zmynd15	2.311957	Susd1	1.517214	Fbxl20	1.27301
Slc15a3	2.298627	Pamr1	1.516791	Usp25	1.272639
4930512H18Rik	2.294978	Gas7	1.515823	Mycbp2	1.272037
A530020G20Rik	2.291215	A230050P20Rik	1.515791	Abca2	1.271611
Abcc3	2.277936	Cd274	1.515474	Ctsb	1.27127
Tap1	2.275735	Gm24187	1.512612	Sfi1	1.271049
Il7	2.274442	Slfn10-ps	1.511997	Capg	1.269608
Micall2	2.266222	Serpinb6b	1.508674	Msi2	1.268826
H2-Ab1	2.255115	H2-M3	1.507956	Adam17	1.267733
Slco3a1	2.249391	Pcdh17	1.507897	2810474O19Rik	1.266183
Ly6c1	2.247399	Pnp	1.506862	Cnp	1.266005
Apol9b	2.243974	Errfi1	1.506135	Rhoj	1.265107
Slfn8	2.237092	Psen2	1.504761	Fbn1	1.265104
Serpina3g	2.223564	Bmper	1.503315	Plekha2	1.264872
Trim30d	2.222229	Rassf2	1.502351	Qsox1	1.264705
Macrod1	2.216982	Rnf150	1.502349	Il4ra	1.262579
Susd6	2.216522	Foxred2	1.502222	Zfp862-ps	1.26213
Rab32	2.208063	Nfkb1	1.500361	Abhd4	1.262
RP24-118K20.1	2.203351	Gm26797	1.499863	Apobec3	1.261504
Islr	2.202374	Cebpb	1.499767	Cryzl1	1.26039
Tnfrsf9	2.201074	Gdnf	1.499677	Snx18	1.259801
Mx2	2.1992	Erap1	1.499261	Snx10	1.259016
Dhx58	2.197596	Phactr1	1.498501	Psme1	1.258257
Mgst2	2.196355	Acy3	1.498313	Prdx5	1.256944
Nlrc5	2.19327	Pde1a	1.497383	Rpl19	1.254924
Ifi27l2a	2.185136	Gm16675	1.4961	Fbxw17	1.254918
Atp8b4	2.184018	Pced1b	1.49569	Ahnak2	1.254603
Dcxr	2.173452	Fndc3a	1.494781	Pgm2	1.254392
Gbp7	2.172556	Sik1	1.48986	Lgals8	1.254212
Nos2	2.172346	Pax5	1.489642	Dusp16	1.254097
Trpc3	2.172073	Rbm47	1.489147	Fdps	1.253066
Col24a1	2.166567	Rhbdf2	1.488124	Zswim4	1.25276
A4galt	2.160477	Gla	1.48683	Tmem9	1.252663
Sp110	2.153144	Mt1	1.486484	Ext1	1.252343
Iigp1	2.150941	Helz2	1.484205	Ldha	1.252201
Bdkrb1	2.144746	Adarb1	1.483657	Ccng1	1.251981
Glrx	2.144381	Manba	1.481685	Rps23	1.251225
Oasl2	2.142329	Ssbp2	1.481023	Traf3	1.251037
Gypc	2.141326	Cd47	1.480263	Tbc1d2b	1.251012
Mark1	2.137578	Gpr176	1.48	Pan2	1.250188
Pdgfra	2.130591	Peli3	1.479421	Ip6k1	1.249537
Tgfbr3	2.126753	Parp11	1.479263	Vegfa	1.248657
Gm20559	2.124888	Agpat9	1.475738	Prrx1	1.248548
Tnfaip3	2.124389	Clip1	1.475604	Nfe2l1	1.247093
Ifit1bl2	2.121353	Pcx	1.475162	Ago1	1.246536
Il6ra	2.115453	Mov10	1.475059	Fgfr1op	1.246307
Cyp7b1	2.114831	Mvp	1.473965	Tnfaip8	1.245604
H2-T22	2.112472	Vdr	1.473286	Appl2	1.245541
Tlr2	2.108417	Ampd3	1.472815	Acaa1a	1.245346
Apol9a	2.107259	Mfsd7c	1.470604	Phip	1.244919
Txnip	2.102973	Ifngr2	1.470338	Rev1	1.244257
Cbr2	2.094355	Nampt	1.47029	Lpin1	1.243059
Ptpn13	2.091855	Stat2	1.469684	Hacl1	1.24284
Isg15	2.084784	Klhl24	1.468335	Abtb1	1.242142
Serpina3f	2.082629	Irak3	1.468302	Zfp281	1.241946
Selp	2.067573	Socs3	1.464374	Pkdcc	1.240434
Gvin1	2.043168	Car11	1.462349	Arhgap12	1.239317
Cmpk2	2.030401	Flt1	1.462052	Malat1	1.23865
Trim12c	2.030228	Ypel3	1.460792	Baiap2	1.236936
Grb14	2.02788	Wdyhv1	1.460703	Sh3d19	1.236615
Gm4841	2.026738	2310001H17Rik	1.458497	Igf2bp2	1.236566
Mnda	2.026163	Slc16a3	1.456228	Fbxo38	1.236407
Igfbp3	2.023331	Cdon	1.455779	Zswim6	1.235488
Gm9574	2.0163	3-Mar	1.455144	Rnf115	1.235458
Tgtp1	2.013841	Psmd10	1.454387	Ubr4	1.233944
Ly6e	1.998818	Cntnap1	1.452562	Calcoco1	1.233454
C4b	1.993132	H2-K2	1.451514	Insr	1.233242
Gfra2	1.985483	Trim25	1.451455	Rps15a	1.233115
Gm2619	1.982496	Scamp1	1.450926	Hexim1	1.233029
Slc39a4	1.982118	Tnfrsf1b	1.449087	Aplp2	1.232116
Osmr	1.97891	Acadsb	1.447878	Ankrd17	1.231804
Ifit1	1.966789	Procr	1.447607	Maff	1.231303
Rrad	1.959059	Pla2g16	1.444745	Foxo4	1.230508
Herc6	1.953162	Atp8a1	1.442774	Urod	1.2304
Clec2d	1.95126	Rbpj	1.441469	Nfib	1.230388
Epas1	1.950642	Neat1	1.440341	Zmynd8	1.229542
9330175E14Rik	1.950302	Il18bp	1.435413	Rsbn1l	1.229463
Lifr	1.947789	Arntl2	1.435176	Mapkapk2	1.228835
Hap1	1.946544	Runx1	1.434923	Lgmn	1.228287
Cfap100	1.939772	BC051226	1.433817	Rasa3	1.228205
Cfh	1.939442	Pvrl2	1.433681	Rps20	1.228122
Slc6a2	1.931558	Zfp874b	1.431978	Chmp4b	1.227714
C1rl	1.930971	Acsl1	1.431187	Prkar2b	1.227104
Abca1	1.922383	Mfsd7a	1.43055	Jun	1.225972
Agrn	1.91988	Mitd1	1.428555	Mmp2	1.225692
Sbno2	1.916865	Ctsh	1.425118	Sumo1	1.225557
Tnip3	1.913463	Zfp874a	1.42474	Tor1aip1	1.225418
Ugcg	1.910687	Mtss1	1.424259	Lacc1	1.225387
Spib	1.907491	Perm1	1.423781	Kdm3a	1.224975
Kcnn3	1.898432	Gsdmd	1.422904	Flnb	1.224811
Ripk2	1.89521	Rspo2	1.422633	Ktn1	1.224573
Ptpn5	1.894565	Gm36936	1.420322	Hspa1b	1.223939
Nod1	1.889504	Dpy19l1	1.419975	Psd	1.223572
Gm4955	1.887092	Spry2	1.418837	Nt5dc2	1.222385
Gm43196	1.885374	Fam3c	1.417203	Usp12	1.222266
Kcnq5	1.876112	Gfpt2	1.4161	Axl	1.222093
Xaf1	1.874808	Ifitm2	1.410051	Akr1b8	1.220794
Lyz2	1.874716	Trim21	1.409367	Gaa	1.219884
C920025E04Rik	1.873125	Pnpla7	1.40874	Ptprj	1.219624
Slc2a6	1.870162	Ociad2	1.408322	Mmab	1.216943
Cxcl16	1.869931	Mkx	1.406854	Osbpl3	1.216658
Foxo3	1.869316	Il10rb	1.406503	Ticam1	1.216655
Relb	1.863299	Vmp1	1.405888	Nub1	1.21658
Ifitm1	1.857611	Spsb1	1.40294	Ogfr	1.216222
Ctps	1.85722	Zfpm2	1.402674	Add3	1.215534
Trim12a	1.851346	Ifi35	1.402586	Slc29a1	1.215147
Ell2	1.849304	Tmem154	1.402112	Nfil3	1.214777
Psmb10	1.849216	Oasl1	1.40142	Parp3	1.21461
Adtrp	1.846019	Irf1	1.401037	Nab1	1.214327
Gm16464	1.843872	Kank1	1.400088	Rpl34	1.214249
Cdk6	1.843573	Traf3ip2	1.399787	Naaa	1.21421
Bnip3	1.840519	Trib1	1.399217	Map1lc3b	1.21098
Plscr1	1.832872	Fbxo32	1.398883	Zfos1	1.210827
Rnf213	1.830593	Dtnbp1	1.398525	Irf9	1.210808
Plscr2	1.824617	Dclk1	1.396242	Vps26a	1.210646
Cgn	1.818312	Gatsl2	1.394958	Col5a3	1.210558
Nek6	1.816836	Irf2	1.39425	Kdm5a	1.209736
Gm43197	1.816332	Dnajc12	1.392043	Tor1aip2	1.209517
P2rx4	1.810723	Ctso	1.391962	Gnptab	1.208163
Rbpms	1.809021	Grina	1.388872	Rab8b	1.207975
Sp140	1.804443	Daam1	1.388466	Spred2	1.207248
Lgals9	1.804377	Cxadr	1.387017	Gdf11	1.206992
Il16	1.803712	Arid5b	1.386992	Pak3	1.206941
Camp	1.801329	Stx11	1.386314	Nlgn2	1.206715
Ube2l6	1.800442	Tcn2	1.385764	Dst	1.206062
Pfkl	1.797849	Ppl	1.38494	Nr1d2	1.205401
Gpr88	1.794428	Aftph	1.383957	Daxx	1.204017
Gm5970	1.793526	Ctsl	1.38209	Uvrag	1.203409
Nfkbie	1.79343	Slc16a1	1.379145	Tnfrsf1a	1.203158
Il20ra	1.793392	B4galt5	1.378838	Cmtm6	1.202344
Rgs16	1.789903	Acvrl1	1.378201	Cstb	1.202249
Ccl9	1.789024	Cx3cl1	1.376893	Il17ra	1.201293
Mettl20	1.78797	Podnl1	1.376677	Stat3	1.200716
Cgnl1	1.781933	Txndc16	1.376549	Sgk1	1.199554
Col6a4	1.781322	Aldh1l1	1.375993	Cldn12	1.197903
Gm19684	1.778757	Crebrf	1.375601	Dync1h1	1.197443
Npc2	1.777478	Ptpre	1.375528	Gabarapl1	1.197238
Igtp	1.776729	Flrt2	1.375148	Tbk1	1.196546
Tapbp	1.776018	Dtwd1	1.374774	Myo18a	1.196491
Slc10a6	1.77264	Il1rl1	1.374317	G3bp2	1.195278
Rtp4	1.772625	Pml	1.373905	Rbm33	1.192694
Itih5	1.77129	Ifit2	1.373384	Eml4	1.192421
Gm12185	1.769756	Rnf114	1.372865	Zmiz1	1.19149
Adhfe1	1.765856	Fth1	1.372822	Psma6	1.19122
Ifnar2	1.760615	H2-T24	1.372506	Csf1	1.189881
Slco1a6	1.753534	Pygl	1.37231	Srsf5	1.1887
Cxcl2	1.752155	Phyh	1.371721	Lmo4	1.187609
Negr1	1.751596	Pik3c2b	1.370604	Pip5k1a	1.182817
Gng2	1.751401	Ttc39c	1.370202	Mlxip	1.181411
Fgf7	1.750411	Myrf	1.369639	Uhrf1bp1l	1.18067
Samd9l	1.750223	Slirp	1.368745	Foxp1	1.175338
Tlr3	1.749767	Mef2a	1.367217	Notch2	1.174766
Tap2	1.73925	Nfkb2	1.366624	N4bp1	1.174303
Irgm2	1.738998	Asah2	1.366535	D17Wsu92e	1.170473
Tifa	1.735914	Ndrg2	1.366351	Prkaa1	1.16978
Tgm1	1.734849	Bnip3l	1.365928	Zc3hav1	1.168237
Birc3	1.728337	Fyco1	1.365796	Abcc1	1.167684
Gm26809	1.725966	Gm6548	1.365763	Paip2	1.164378
Il34	1.725927	Gpr146	1.36386	Bsg	1.161778
Thbs2	1.722317	Plekhn1	1.362388	P4ha1	1.160257
Ppm1k	1.720243	Ghr	1.360096	Pld3	1.160076
Casp12	1.719866	Cnnm2	1.359865	Lamc1	1.159628
Arhgdib	1.719733	Arid5a	1.359164	Ece1	1.15875
Stab 1	1.719107	Car13	1.358995	Dcaf8	1.154963
Nmi	1.7184	Jak3	1.358706	Psap	1.148899
Ptgir	1.71638				


Gene symbol	Fold change	Gene symbol	Fold change	Gene symbol	Fold change
Col2a1	0.194072	AI506816	0.692198	Zfp36l2	0.779391
Megf6	0.2661	Coro2b	0.692611	Uchl1	0.780148
Col11a2	0.305052	Mybl2	0.692634	Mageh1	0.780348
Pdlim3	0.320795	Ckb	0.693344	Pear1	0.780434
Hes1	0.358613	Ltbp4	0.693767	Chd3	0.780444
Cnn1	0.360323	Col6a3	0.694023	Tmem214	0.780529
Chodl	0.361883	Lrp4	0.694203	Ehbp1l1	0.780809
Cthrc1	0.362334	Gli2	0.694925	Ncapg2	0.781412
Col27a1	0.365089	Osbpl10	0.695198	Vldlr	0.781545
Adamts18	0.373978	Kif18a	0.697441	Ajuba	0.781571
Alcam	0.3757	Birc5	0.697479	Cad	0.781967
H19	0.376363	Klhdc8a	0.69799	Crip2	0.782054
Cmklr1	0.398128	Kif14	0.698154	Lnp	0.783162
Acta2	0.406436	Ttll3	0.698273	Tmsb10	0.783219
Igfbp2	0.40714	Nid2	0.698295	Plaur	0.783269
Kirrel3	0.412516	4930427A07Rik	0.698662	Kctd11	0.783405
Crlf1	0.415332	Spc24	0.698751	Fam160a2	0.783821
Adamtsl1	0.417672	Pcsk9	0.69891	Mis18bp1	0.783968
Egr3	0.429728	Tmem144	0.6995	Cenpq	0.784621
Kcnh2	0.431717	Cenpp	0.699524	Mcm8	0.784843
Fam132b	0.437393	Bmp5	0.699542	Nucks1	0.784887
AI593442	0.43824	Aurka	0.700218	Sec23a	0.78531
Slc14a1	0.438322	Radil	0.700268	Plxna1	0.785572
Lrrc17	0.439491	Espl1	0.701332	Troap	0.786395
C1qtnf3	0.44141	Crabp1	0.701809	Rraga	0.786476
Dock8	0.443083	Tenm3	0.702765	Spdl1	0.78654
Hr	0.446347	Adm2	0.702837	Klf13	0.786594
Pappa2	0.45507	2810417H13Rik	0.702964	Cdk1	0.786628
Bok	0.455099	Cnn2	0.703147	Igfbp6	0.786699
Sorbs2	0.455931	Ska3	0.703358	Svil	0.786846
Tspan18	0.457162	Tubb5	0.705065	Shf	0.787274
Fgf18	0.46685	Cenpe	0.705359	Paqr4	0.787633
Eln	0.468291	Slc24a3	0.705444	Ids	0.788248
Dlx5	0.46936	Plekhh3	0.706342	Fkbp10	0.788316
Heyl	0.472756	Parvb	0.706447	Sun2	0.788366
Adra1d	0.476707	Fbln5	0.706969	Pbx3	0.788441
Cacna1h	0.482864	Adra1b	0.707729	Mthfd2	0.788495
Fam101b	0.490039	Flna	0.708151	Lrrc59	0.788518
Thbd	0.490796	Enc1	0.708306	Ticrr	0.788655
Epha1	0.496545	Tmeff2	0.708497	Gata2	0.788815
Actg2	0.500303	Mdga1	0.708616	Tiam2	0.788818
Ccser1	0.502486	Fbxo5	0.708739	Yif1b	0.789025
Sema3a	0.503289	4930503L19Rik	0.708843	Myh9	0.789258
Rnf128	0.50426	Cit	0.709144	Srm	0.789413
Glis1	0.50534	Igsf9	0.709326	Hmgb1	0.789802
Pparg	0.507961	Crip1	0.709588	Arf2	0.789924
Dpysl3	0.511253	Mad2l1	0.70974	Fancd2	0.790044
Plppr4	0.5121	Fblim1	0.709755	Smc2	0.790137
Aqp1	0.512552	Dck	0.709924	Nrp1	0.790629
Flrt3	0.514301	Chpf	0.710145	Fes	0.790664
Acan	0.514645	Tmeff1	0.71038	Herpud1	0.790702
Fbln7	0.515943	Unc5b	0.71092	Myh10	0.791068
Perp	0.516446	Cdca8	0.710994	Tmem200b	0.791145
Trpv2	0.516591	Pycr1	0.711174	Copz2	0.791267
Wscd2	0.51676	Naaladl1	0.711837	Ube2s	0.791564
Elmo1	0.517566	Tusc1	0.711918	Wdr1	0.79158
Ube2ql1	0.517989	Slc18b1	0.712161	Cdkn2c	0.791604
Hey1	0.518071	Plagl1	0.713162	Alyref	0.791762
Cdh3	0.51819	Melk	0.713693	Clmn	0.791941
Tagln	0.522316	Cdc25b	0.714193	Dlc1	0.791964
Mest	0.522663	Baiap2l1	0.714203	Mesdc1	0.791976
Pcolce2	0.523521	St3gal6	0.714705	Midn	0.792069
Cyp26b1	0.523717	Col16a1	0.714714	Megf9	0.792474
Pdgfc	0.523729	Hoxb5	0.714759	Cep41	0.79254
Irx3	0.525344	Phf19	0.714806	Npepl1	0.792581
Fmod	0.525701	Oxct1	0.714916	Ptrf	0.792717
Itga8	0.529252	Fam171a1	0.716872	Kctd9	0.793202
Erg	0.529291	Pcdh19	0.717354	Crtap	0.793212
Nfatc2	0.531646	Stbd1	0.717433	Plppr3	0.793224
Adamts16	0.531726	Fhod3	0.717683	Rusc2	0.793289
Palmd	0.531982	Basp1	0.717717	Adam19	0.793359
Foxd1	0.53212	Anln	0.717824	Akap12	0.793685
Wnt2b	0.532995	Nek2	0.71792	Snhg5	0.793858
Limch1	0.533269	Phgdh	0.718101	Dbf4	0.793865
Adgrl3	0.533541	Cacna1c	0.718104	Fig4	0.794195
Cd24a	0.536601	Hmmr	0.718652	Tmcc2	0.794436
Gpc1	0.537676	Chac1	0.718843	Mcm10	0.794634
Itga11	0.538779	Palld	0.718898	Akirin2	0.794761
Gprc5c	0.53903	Sgol1	0.719211	Ppp1r13l	0.795115
Frem1	0.541975	Ska1	0.719223	Sorl1	0.795125
Plcl1	0.5431	Tmsb4x	0.719624	Aif1l	0.795392
Col15a1	0.546367	Irs1	0.719744	Tacc2	0.795702
Fhl1	0.547264	Spag5	0.719865	Rc3h2	0.795776
Mfap4	0.548733	Acot2	0.719927	Cdc25c	0.795811
Mcam	0.54916	Cenpl	0.720249	Olfml2b	0.796074
Gm14321	0.549468	Kif20a	0.720582	Vasn	0.796149
Hmgb3	0.552312	Dlx1	0.720615	Bub1	0.796335
Smoc2	0.552559	Lama2	0.720683	Xrcc1	0.796544
Thbs1	0.553418	Glis2	0.72081	Shmt2	0.797154
Ptn	0.555662	Kif4	0.72101	Sertad3	0.797265
Igf1	0.557068	P3h1	0.72114	Anxa2	0.797684
Fbn2	0.558778	Pdlim7	0.721444	Tubb4b	0.79815
Card10	0.560923	Angptl2	0.72184	Nab2	0.798326
Gm43719	0.561666	Id1	0.721848	Cryab	0.798375
Gm17315	0.561884	Sorcs2	0.722051	Fzr1	0.798917
Lrrc75b	0.562648	Kif26b	0.722251	Alg8	0.798963
Kcnk6	0.565036	Dars2	0.72227	Zbtb14	0.799177
Rnf39	0.565946	Stk39	0.722641	Pcolce	0.799313
Pcp4l1	0.565995	2700099C18Rik	0.722713	Rara	0.799377
Lims2	0.568426	Tpx2	0.7228	S100a10	0.799605
Rxfp3	0.568433	Nusap1	0.723138	Taf5	0.799838
Mb21d2	0.56918	Cald1	0.723271	Col5a1	0.800168
Id4	0.569201	Cenpn	0.723906	Ercc6	0.800219
Pitx2	0.569411	Cenpi	0.724017	Exo1	0.800893
Sox5	0.569607	Lrr1	0.724263	Myadm	0.801324
Ahrr	0.571186	Kifc5b	0.724644	Ggcx	0.801779
Efs	0.571392	Kif22	0.725384	Klf16	0.801989
Sema3d	0.571535	Cenpm	0.725625	Gpaa1	0.802171
Etl4	0.573284	Ptprk	0.725655	Pmf1	0.802196
Megf10	0.573779	Syk	0.725715	D030056L22Rik	0.80231
Arhgap36	0.573854	Gm22	0.725943	Grk6	0.802539
Hbegf	0.575347	Rsph3b	0.726129	Phlda3	0.802648
Pi15	0.576538	Tanc1	0.7262	Ddah1	0.802713
Ank1	0.577911	Hid1	0.726245	Cd248	0.803109
Lrrn3	0.578392	4933404O12Rik	0.726327	Rcn1	0.80311
Tmem238	0.578485	Ap1s2	0.726428	Got1	0.803556
Tcp11l1	0.579068	Haus8	0.726684	Slc6a6	0.803585
Ccna1	0.579425	Ncapd2	0.727616	Prx	0.803637
St8sia2	0.579469	Marcks	0.727633	Map1s	0.803739
Aoc3	0.579637	Tgfb3	0.727733	Wwp2	0.804876
Prr7	0.579853	Fzd8	0.728304	Pkd1	0.8053
Tspan2	0.580597	Eya4	0.728318	Pithd1	0.805306
Plekhg1	0.58319	Cxxc5	0.72853	Iqgap3	0.805603
Tnfrsf11b	0.583534	Mn1	0.728747	Tbl1xr1	0.805732
Gm9936	0.584827	Cenpa	0.728756	Ddx39	0.805924
Gper1	0.584873	Hcfc1r1	0.72942	Capn2	0.80608
Ctgf	0.586173	Itgbl1	0.729729	Edem1	0.806473
Psrc1	0.586316	Ccna2	0.729782	F3	0.80656
Mxd3	0.586498	Reep4	0.729883	Gltp	0.806717
Npas4	0.587419	Pck2	0.730548	Ckap2	0.806725
Dlg2	0.587439	Ank	0.730673	Slc3a2	0.806799
9230111E07Rik	0.590223	Cdca2	0.730751	Ado	0.806898
Ano1	0.590542	Sim2	0.730981	Slc35a2	0.807269
Gas2	0.59414	B3gnt9	0.731156	Cntrob	0.807304
Rtn4r	0.596076	Top2a	0.731442	Mmp14	0.807447
Col12a1	0.597466	Cdc42ep3	0.731593	Arhgap5	0.807549
Gm26737	0.597617	Kif18b	0.732018	Zfp827	0.807876
Adcy1	0.597814	Syde2	0.732501	Trim59	0.80795
Gm2115	0.598004	Ctnnal1	0.732705	Cyp20a1	0.808079
Dgkg	0.598166	Daam2	0.733135	Plxnb1	0.808099
Sox9	0.598415	Depdc1a	0.7333	Tcaf1	0.808114
Map3k7cl	0.599708	Trpv4	0.733578	Adamts5	0.808125
Prkg1	0.599718	Dstn	0.733957	Slc12a2	0.808408
Ankle1	0.599936	Wisp1	0.734367	Txndc5	0.808421
Otud1	0.600065	Tpm2	0.734462	Trim27	0.808773
Jag1	0.600976	Ect2	0.734528	Ptgfrn	0.808829
2700069I18Rik	0.601158	Gmppb	0.734658	Rbm15b	0.808867
Dhrs3	0.601739	Mastl	0.734793	Gnas	0.809065
Slc40a1	0.603454	Tgfb1i1	0.735408	Plp2	0.809371
1700061I17Rik	0.604504	Myo1e	0.73552	Casc5	0.80943
Cap2	0.604809	Stmn1	0.735545	Prkcdbp	0.809663
Ctnnd2	0.606786	Odf2	0.735631	Ndc1	0.809727
Gas6	0.607571	Bub1b	0.736395	Cyb5r1	0.810306
Rcan2	0.607984	Gm1976	0.736422	Haus7	0.810536
Vim	0.608469	Racgap1	0.736603	Hjurp	0.810706
Myl9	0.609077	Gpsm1	0.736621	Cd34	0.810768
Pif1	0.609688	Gm12715	0.73667	Alg5	0.810783
Ptprv	0.609772	Kifc1	0.736797	S100a11	0.810974
Vav3	0.610392	Fabp5	0.737278	Tk1	0.811112
Myh11	0.610733	Notch1	0.737474	Sertad2	0.811164
C430049B03Rik	0.610924	Rnf26	0.738216	Smarcd1	0.811398
Irx5	0.611034	Actg1	0.738473	Mbnl1	0.811622
Cspg4	0.611366	Poc5	0.738631	9430015G10Rik	0.812019
Jazf1	0.611816	Fhl2	0.738831	Lgals1	0.812145
Npr3	0.612709	Aspm	0.7396	Gars	0.812233
Nog	0.614075	Mettl1	0.739674	Map6	0.812248
Crispld2	0.614285	Oip5	0.739925	Dhx57	0.812313
Htra3	0.615126	Lmnb1	0.74011	Fnbp1l	0.812351
Kazald1	0.615545	Fgfr2	0.740416	Uevld	0.812642
Smad9	0.616235	Prc1	0.740456	Klhdc10	0.812852
Gata3	0.616758	Aurkb	0.740768	Ybx3	0.813191
Fam198b	0.617949	Slc7a5	0.740978	Tmem263	0.813199
Prex2	0.619588	Gsg2	0.741532	Tes	0.813433
Itgb3	0.621091	Ptx3	0.741631	Gas2l3	0.813511
Pcsk6	0.621802	Nuak1	0.742034	Esyt1	0.813751
Adamts1	0.622628	Lockd	0.742214	Hmgn2	0.814175
Fam131b	0.623292	Nuf2	0.7423	Anxa5	0.814195
Grb10	0.623477	Col8a2	0.742911	Kdelr3	0.814222
Ascl1	0.623525	Odc1	0.742989	Klf10	0.81458
Ptprm	0.624038	Pxdc1	0.743054	C77080	0.814703
Chst2	0.624537	Cep55	0.743097	Lmf2	0.81475
Gli1	0.624862	Tuba1a	0.743583	Cbarp	0.815046
Rtn4rl1	0.624873	Shcbp1	0.743698	Plekhg2	0.815326
Arxes2	0.627232	4931428F04Rik	0.743798	Clcn2	0.816112
Cldn15	0.627251	St6galnac2	0.743933	Eif1	0.816272
Map2	0.627453	Recql4	0.743985	0610009O20Rik	0.816622
A730020E08Rik	0.627473	Masp1	0.743993	Kpna2	0.816748
Paqr8	0.628133	Rhob	0.745098	Emp1	0.816779
Slc2a10	0.628161	Ldlrad3	0.745422	Fndc4	0.816887
Fam83d	0.628566	Gm11223	0.745634	Fhod1	0.816915
Sema6d	0.62888	Cdkn3	0.745643	Fam149a	0.817229
Rltpr	0.629271	Aff3	0.745775	Tnrc18	0.817407
Inhba	0.629286	Cplx2	0.745867	C330027C09Rik	0.817453
Tgfbi	0.631807	Nfix	0.745916	Sec24d	0.817484
Gcnt4	0.632013	Taf4a	0.74597	Wsb2	0.817526
Mertk	0.632108	Cdkn2d	0.746083	Dda1	0.817574
C1ql3	0.633177	Pfn1	0.746768	Rtel1	0.818013
Lpar3	0.633363	Srf	0.747411	Prep	0.81832
Wnt11	0.633839	Aldh1l2	0.748636	Sec24a	0.818454
Gpc6	0.634565	Cdca5	0.748855	Soga1	0.818645
Shank2	0.634576	Dlgap5	0.748977	Obsl1	0.818749
Fam65b	0.634732	Gpt2	0.749626	Sox4	0.819024
Optc	0.634952	Plxdc2	0.749849	Olfml3	0.819129
Cldn1	0.635369	Fam20c	0.750155	Dpysl2	0.819636
Ucp2	0.635725	Dolpp1	0.750377	Arpc5	0.819906
Tub	0.636665	Msrb3	0.75087	Mdm1	0.820017
Esr2	0.637654	Gpx7	0.751128	Maged1	0.820067
Zcchc5	0.637808	Gpc4	0.751191	Efemp2	0.820203
Cgref1	0.637844	Sh3rf3	0.751228	Maz	0.820264
Cobl	0.637877	Mical1	0.75133	Rcc1	0.820373
Efcab11	0.639182	Plk4	0.751777	Limk1	0.820457
Clec11a	0.639468	Id2	0.751969	Sdc1	0.820495
Mfap2	0.640436	Svep1	0.752872	Pfas	0.820519
Fam64a	0.640806	Depdc1b	0.75297	Bora	0.820562
Agtr2	0.64086	Tsc22d3	0.753152	2700081O15Rik	0.820759
Aspn	0.641139	Tuba1c	0.75324	Degs1	0.820789
Cdca3	0.641611	Ccnb1	0.753654	Slc7a1	0.82085
Gm26651	0.642262	B4galt2	0.753667	Ckap5	0.821241
Rrm2	0.642632	Tpm1	0.753828	Dennd2a	0.821364
Fam122b	0.642715	Rian	0.75385	Marveld1	0.821535
Fgf21	0.643125	Gramd2	0.753866	Ncaph	0.822363
L1cam	0.643173	Ttyh3	0.754083	Maf	0.822728
Prss23	0.643878	Depdc7	0.754102	Arid1b	0.822833
Nrep	0.644	Hyls1	0.754994	Pitrm1	0.822917
Srgap1	0.644061	Adamts6	0.755142	Ran	0.823
Dusp5	0.644116	Dap	0.755702	Nfyb	0.823101
Lonrf1	0.645283	Pkmyt1	0.755798	E2f1	0.823463
Artn	0.6468	Zdhhc2	0.755898	Dpp9	0.82356
Slc17a9	0.646801	Irs2	0.756009	H2afz	0.823848
Nexn	0.647017	Larp1b	0.756428	Gmnn	0.823908
Efnb2	0.648037	Trp53i13	0.756504	Nfatc4	0.823922
C530008M17Rik	0.648087	Tmpo	0.756673	Bmi1	0.823945
E2f8	0.648404	Tmcc3	0.757004	Anapc16	0.824058
Adrb1	0.649094	Cdk2ap1	0.757041	Fscn1	0.824548
Snai1	0.649563	Mtss1l	0.75718	Lmna	0.824985
Gja1	0.650241	Mum1l1	0.757403	Mex3c	0.825829
Cilp2	0.651178	Pbk	0.757669	Rad51	0.825881
Kitl	0.651609	Tcf7l2	0.758116	Sepn1	0.825965
H1fx	0.651903	Fam8a1	0.758148	Csrp1	0.826069
Vit	0.652032	Plk1	0.758271	mt-Nd2	0.826147
Shank1	0.652795	Hccs	0.758344	Specc1	0.826198
Lmod1	0.653332	Nde1	0.758371	Gsr	0.826224
Tacc3	0.653646	Mki67	0.758515	Osbp	0.826319
E2f7	0.653948	Ccnf	0.758729	Net1	0.826663
Trim66	0.654695	Ttl	0.759024	Mpp1	0.826811
Sapcd2	0.654882	Col1a2	0.75928	Ehd2	0.827066
Hoxa10	0.655107	Spire2	0.759392	Oaf	0.82721
Gamt	0.655282	Trip13	0.759459	Tcf19	0.827416
Neurl1b	0.655678	Gm17501	0.759486	P3h4	0.827463
Ntn4	0.656116	Coq10b	0.760732	Col5a2	0.827588
Zfp804a	0.656484	Zfp9	0.760978	Ncs1	0.827762
Plau	0.657035	Thy1	0.761234	Thbs3	0.828031
Lingo1	0.657449	Fgfrl1	0.761271	Dad1	0.828311
Ctxn1	0.657571	Incenp	0.761488	Hmces	0.828493
Omd	0.657621	Itga1	0.76159	Eef2k	0.828914
Kif23	0.657802	Fkbp14	0.762018	Serpinh1	0.830267
Spta1	0.657819	Col6a2	0.762075	Cenpc1	0.830422
Arhgap19	0.658013	Cks2	0.762688	Dna2	0.831046
Fzd6	0.6582	Zfp518b	0.763119	Adgrl1	0.831229
Hic1	0.658269	Vcl	0.763179	Pom121	0.831379
Chtf18	0.658417	Sgol2a	0.763283	Dtymk	0.831805
Trib3	0.65844	Cenpu	0.763296	Elavl1	0.832095
Prkab2	0.658741	Zbed3	0.763309	Mcm5	0.832182
Rps6ka2	0.658892	Yrdc	0.763508	5-Sep	0.83248
Prr5l	0.658974	Pask	0.763629	Col4a2	0.833235
Tcf7l1	0.659331	Ska2	0.763681	Mpp6	0.834295
Foxm1	0.659418	Cdkl2	0.763788	Gclc	0.834364
Col1a1	0.659671	Dock5	0.764101	Ppp2r5a	0.835098
Sh3rf2	0.65978	Scarf2	0.764653	Zw10	0.835134
Cdh1	0.660507	Ncapg	0.765	Met	0.835261
Smtn	0.661819	Tmem119	0.765155	Clspn	0.835293
Lmo1	0.662257	Fgfr3	0.765166	Kntc1	0.83531
Fam110b	0.663262	Neil3	0.765235	Plpp5	0.83564
Slc7a3	0.66339	Dact3	0.765316	Pygb	0.835725
Ube2c	0.663546	Pusl1	0.765544	Ino80e	0.835727
Cdh2	0.664276	Kif2c	0.765579	Myo1c	0.836357
Cdc20	0.664437	Mtus1	0.76598	Fam114a1	0.836547
Serinc2	0.664934	H2afx	0.766012	Nup85	0.836806
Zyx	0.665352	Klhl23	0.766603	Emx2	0.837083
Wnt2	0.66692	Sh3bgrl3	0.766626	Tcof1	0.837375
Timp3	0.667381	Prrc1	0.766849	Eif2ak3	0.838006
Olfm1	0.667483	Gcsh	0.767235	Fez2	0.838275
Lhx9	0.667998	Slc1a4	0.767371	Qk	0.838602
Nqo1	0.669185	Fat4	0.767491	Pmp22	0.838661
Ndc80	0.669493	Gm14005	0.767686	Tnc	0.839473
Foxc2	0.669981	Kdelr2	0.76815	Golim4	0.839552
Ccdc80	0.670023	Qpct	0.768216	Gpsm2	0.839794
Tmem158	0.670685	BC030867	0.768468	Adgrg6	0.839888
Jup	0.67169	Efnb1	0.76946	Rpa2	0.840289
Hmcn1	0.671695	Kif20b	0.769555	Ckap4	0.840779
Esco2	0.67216	Creb3l1	0.76961	Col4a1	0.842209
Lfng	0.672375	Ifi27	0.769882	Impad1	0.842282
Gtse1	0.672472	Myl6	0.769907	Nras	0.842384
Ckap2l	0.673922	Zfp354c	0.769947	Mxd4	0.842803
Col11a1	0.673993	Actb	0.769987	Nup107	0.842931
St6gal1	0.674366	Pmepa1	0.770031	Kpnb1	0.843093
Egr2	0.674946	Cenpt	0.770107	Tcf4	0.843201
Ccdc85c	0.67579	Nfatc1	0.770872	Mthfd1l	0.843236
Adamtsl3	0.676597	Pcyt1b	0.771831	Klf6	0.843283
Amot	0.67673	Pofut2	0.772115	Ikbip	0.844251
Parpbp	0.676812	Tmem64	0.772532	Egr1	0.844545
Rps4l	0.676948	Celf2	0.772687	Pcbp1	0.845333
Piezo2	0.677444	Fgd1	0.772758	Arfgap1	0.845823
Pkp1	0.67865	2700094K13Rik	0.772841	Fermt2	0.845996
Ezr	0.67867	Rbmx	0.772952	Fkbp9	0.846524
Ccdc169	0.679208	Rangap1	0.772989	Pofut1	0.84685
Slc43a1	0.679473	Pkp4	0.772993	Ptbp1	0.847263
Prune2	0.679475	Anapc11	0.773243	Copg1	0.848294
Kcp	0.68011	Anxa6	0.773856	Kif1c	0.848401
Dysf	0.680215	Ccnb2	0.774255	Arpc4	0.848799
Cav1	0.680273	Dzip1l	0.774278	Creb3l2	0.851804
Miip	0.680407	Aldh18a1	0.774618	Lamb2	0.85188
Cenpf	0.680683	Ccdc18	0.774722	Cbx5	0.851952
Adamts12	0.680685	Arhgap11a	0.775249	Actn1	0.852875
Asf1b	0.681309	Slc6a9	0.775406	Cd9	0.853406
Lgr6	0.681438	Siah2	0.775723	Wwc2	0.854263
Dlx2	0.683214	Ccdc74a	0.775994	Gls	0.854739
Doc2b	0.684296	P4ha3	0.77607	Sfr1	0.85486
Gulp1	0.685434	Dusp7	0.776231	Rcn3	0.855116
Hacd1	0.685474	Slc27a3	0.776587	Pias3	0.855486
Plcb1	0.6858	Atf5	0.776605	Kctd10	0.855905
Dnm3os	0.686603	Pde3b	0.776666	Cntrl	0.855999
Arnt2	0.688141	Garnl3	0.777086	Cs	0.856556
Prelp	0.688287	Bcl2l1	0.777251	Maged2	0.858124
Cdr2	0.68853	Fhl3	0.777932	Atp1a1	0.858214
Morc4	0.689027	Kif15	0.778861	Golga2	0.859759
Tcaf2	0.690546	Rad51ap1	0.778895	Lbr	0.860158
Spc25	0.690558	Adgre5	0.778903	Setd8	0.860806
Kctd15	0.690753	Brca1	0.779076	Slc35e1	0.8627
Arhgap33	0.690899	Vangl1	0.779078	Uhrf1	0.86398
Kif11	0.690918	Tuba1b	0.779294	Calu	0.868509
Six2	0.691737	Atf4	0.779332	Crim1	0.869402
Glt8d2	0.692094	Ttk	0.779355		

Pulmonary TCs were reported to promote angiogenesis in a mouse model of ARDS [11]; thus, particular attention was devoted to angiogenesis in the gene ontology (GO) functional analysis. According to the DAVID online database, 28 DEGs were enriched in the processes of blood vessel formation, angiogenesis, blood vessel morphogenesis, blood vessel remodelling, and sprouting angiogenesis. For further analysis, the DEGs were enriched in the STRING database. According to the STRING database, the DEGs were enriched in 9 angiogenesis-related processes: angiogenesis, blood vessel morphogenesis, vasculature development, blood vessel remodelling, sprouting angiogenesis, venous blood vessel sprouting, venous blood vessel morphogenesis, regulation of angiogenesis, and positive regulation of angiogenesis (Fig. 3d). As most genes participated in at least 3 biological processes, those involved in more than three processes—i.e. E2F8, Notch1, EPAS1, Rbpj, Flt1, ACVRL1, EFNB2 and Thbs1—were selected for further research. MiR-21a-3p, miR-221-5p, miR-146a-5p and miR-188-5p regulated these 8 genes (Fig. 3e).

### Validation of miRNAs and their target mRNAs in TCs

**Fig. 4:**
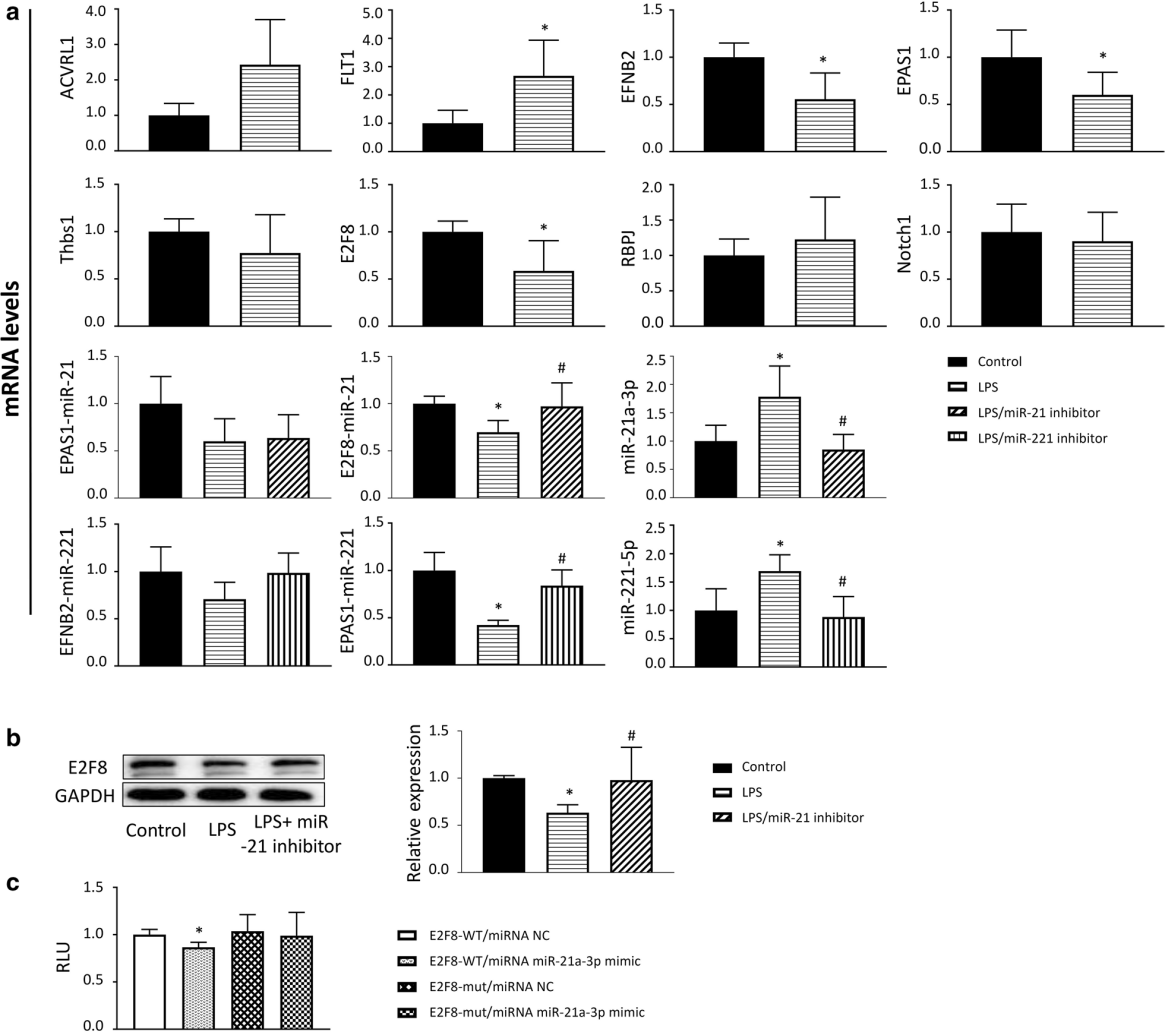
Expression of angiogenesis-related genes in TCs with LPS treatment. **a** mRNA levels of E2F8, Notch1, EPAS1, Rbpj, Flt1, ACVRL1, EFNB2, Thbs1, FLT1, miR-21a-3p and miR-221-5p in TCs treated with LPS and/or miR-21a-3p inhibitor or miR-221-5p inhibitor. *P < 0.05 vs Control, ^#^*P *< 0.05 vs LPS. **b** Protein levels of E2F8 in TCs treated with LPS and/or miR-21a-3p inhibitor. *P < 0.05 vs Control, ^#^*P* < 0.05 vs LPS. **c** MiR-21a-3p led to a significant reduction of the luciferase activity of reporter with the wildtype 3′ UTR but not that of the mutant reporter. *P < 0.05 vs E2F8-WT/miRNA miR-21a-3p mimic. n = 6

We next assessed the mRNA levels of angiogenesis factors. The mRNA expression of E2F8, Notch1, EPAS1, Rbpj, Flt1, ACVRL1, EFNB2 and Thbs1 was measured in TCs after LPS stimulation. After LPS stimulation, E2F8, EFNB2, and EPAS1 were significantly downregulated, while Flt1 was upregulated. Given that miRNAs usually negatively regulate downstream genes, E2F8, EFNB2, and EPAS1 were further studied. LPS stimulation significantly increased miR-21a-3p and miR-221-5p expression in TCs compared with that in cells under control conditions. To clarify the relationship between miRNAs and mRNAs, miRNA inhibitors were applied. MiR-221-5p inhibition restored the expression of EPAS1 but not EFNB2, and miR-21a-3p inhibition restored the expression of E2F8 but not EPAS1. MiR-21 had been reported to increase proliferation, migration and tube formation of Human Umbilical Vein Endothelial Cells (HUVECs) and induce angiogenesis by directly targeting PTEN [30, 31]. Moreover, miR-21a-3p and its downstream target E2F8 were further studied. After 24 h, the protein expression of E2F8 was decreased in TCs challenged with LPS and was restored by inhibition of miR-21a-3p. The dual luciferase reporter assay indicated that E2F8 was the direct target of miR-21a-3p (Fig. 4).

### MiR-21a-3p regulated angiogenesis under inflammatory conditions

**Fig. 5:**
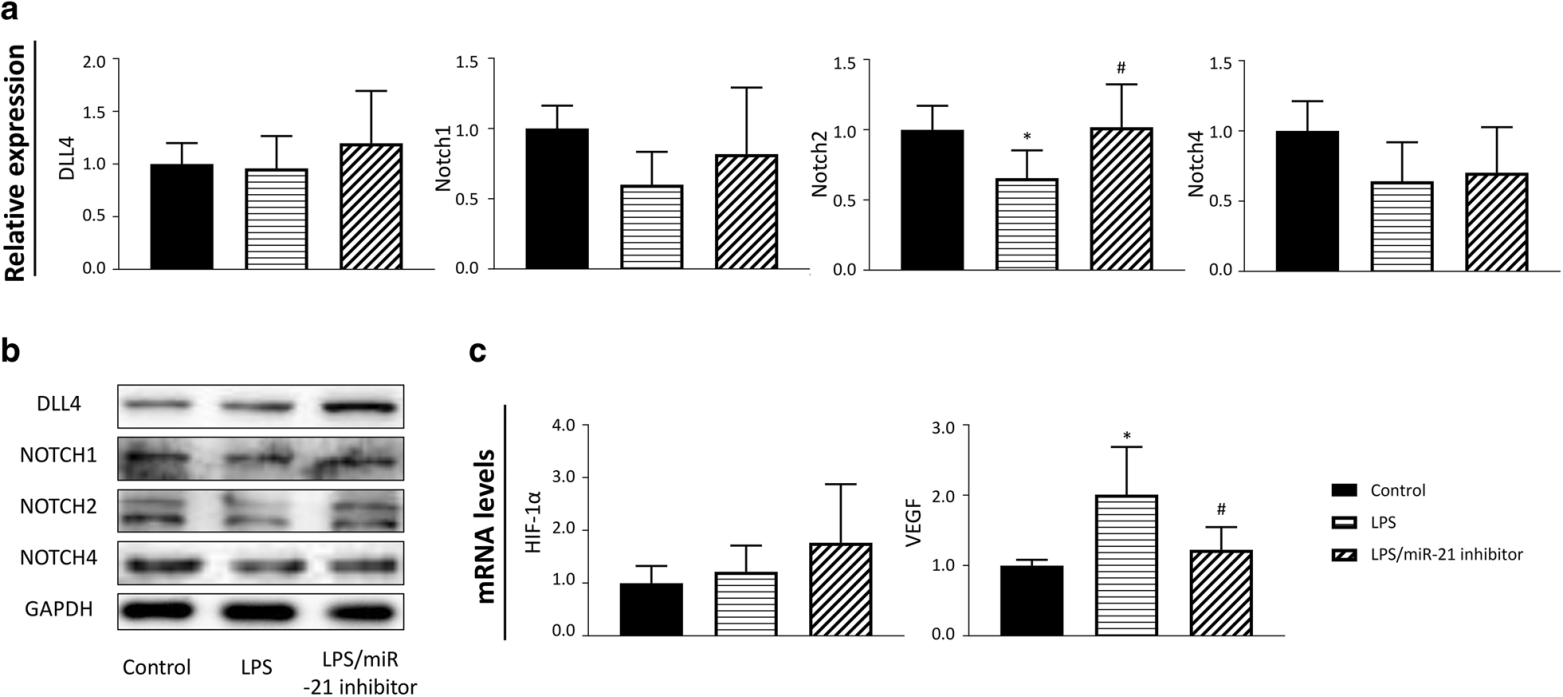
MiR-21a-3p regulated angiogenesis associated signalling in TCs induced with LPS. **a**, **b** Protein levels of DLL4, Notch1, Notch2, and Notch4 in TCs treated with LPS and/or miR-21a-3p inhibitor. **c** Expression of HIF-1α and VEGF on mRNA level. *P < 0.05 vs Control, ^#^*P* < 0.05 vs LPS, n = 6

The transcription factors E2F7/8 were reported to regulate vessel branching via DLL4-Notch approaches [32] or HIF-1α/VEGFA signalling [33]. In the present study, LPS stimulation reduced the protein expression of Notch 2 but not Notch 1, Notch 4 or DLL4. Inhibition of miR-21a-3p restored Notch 2 protein expression in TCs in the presence of LPS. LPS did not affect HIF-1α expression. However, LPS increased the expression of VEGFA at the mRNA level, and this increase was reversed by miR-21a-3p inhibition in cultured TCs (Fig. 5).

### PI3K signalling might participate in angiogenesis

**Fig. 6:**
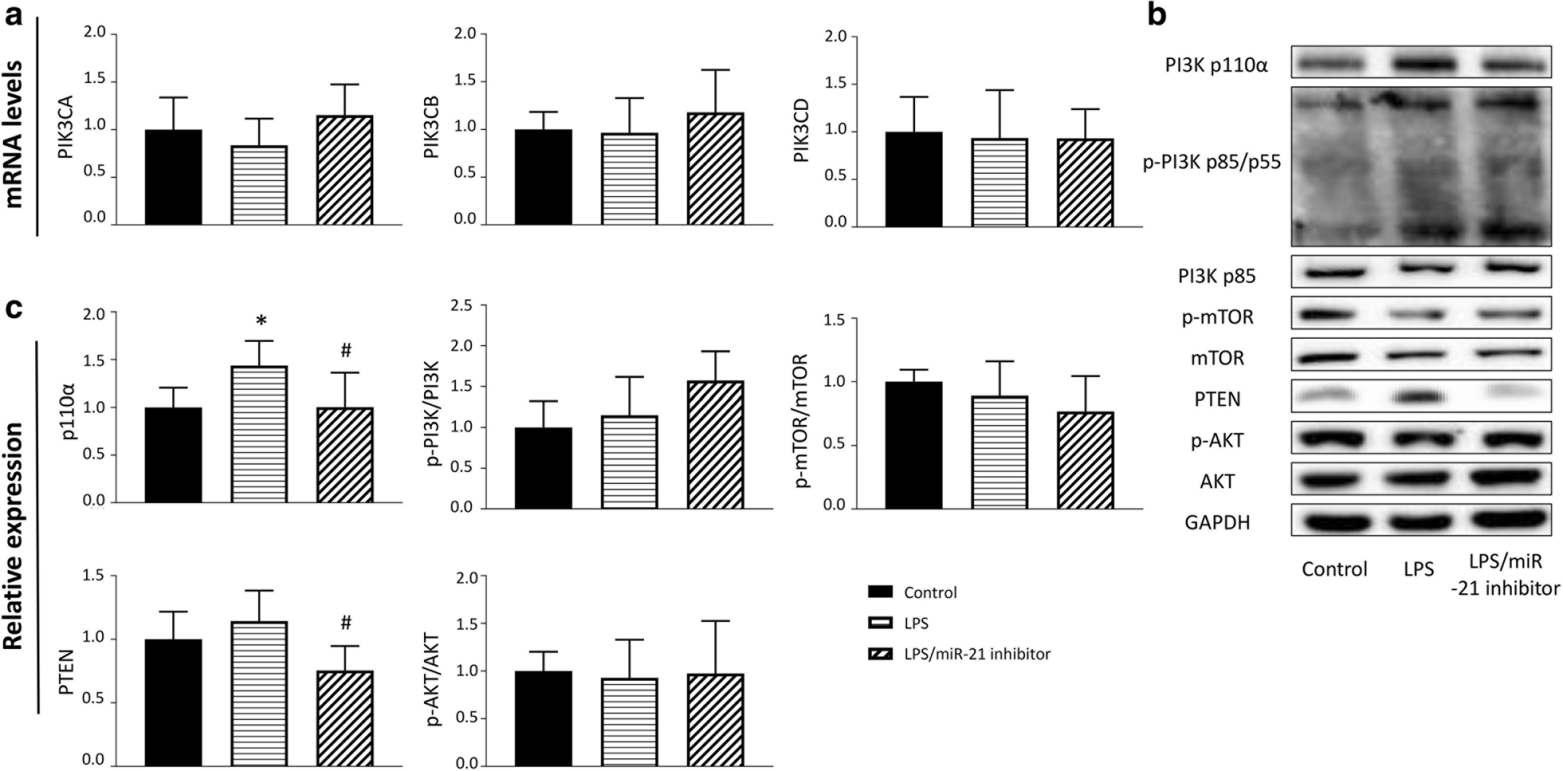
Expression of PI3K signalling in TCs induced with LPS. **a** mRNA levels of PIK3CA, PIK3CB, and PIK3CD in TCs treated with LPS and/or miR-21a-3p inhibitor. **b**, **c** Protein levels of p110α, p-PI3K, PI3K, p-mTOR, mTOR, PTEN, p-AKT, AKT in TCs treated with LPS and/or miR-21a-3p inhibitor. *P < 0.05 vs Control, ^#^*P* < 0.05 vs LPS, n = 6

PI3K, especially the Class I catalytic isoforms, plays an important role in angiogenesis. To study the mechanisms underlying the effect of miR-21a-3p in TCs on angiogenesis induction, PI3K subunit expression was first examined. The mRNA levels of the Class I PI3K isoforms PIK3CA, PIK3CB, and PIK3CD did not significantly change with LPS stimulation. However, the protein level of p110α in TCs was significantly increased with LPS stimulation and decreased with miR-21a-3p inhibitor co-treatment. The PI3K signalling molecules AKT, mTOR, and PTEN were unaffected by either LPS or miR-21a-3p. These results indicated that PI3K signalling might participate in angiogenesis via the p110α isoform (Fig. 6).

### MiR-21a-3p and p110α in TCs promoted the proliferation of EOMA cells

**Fig. 7:**
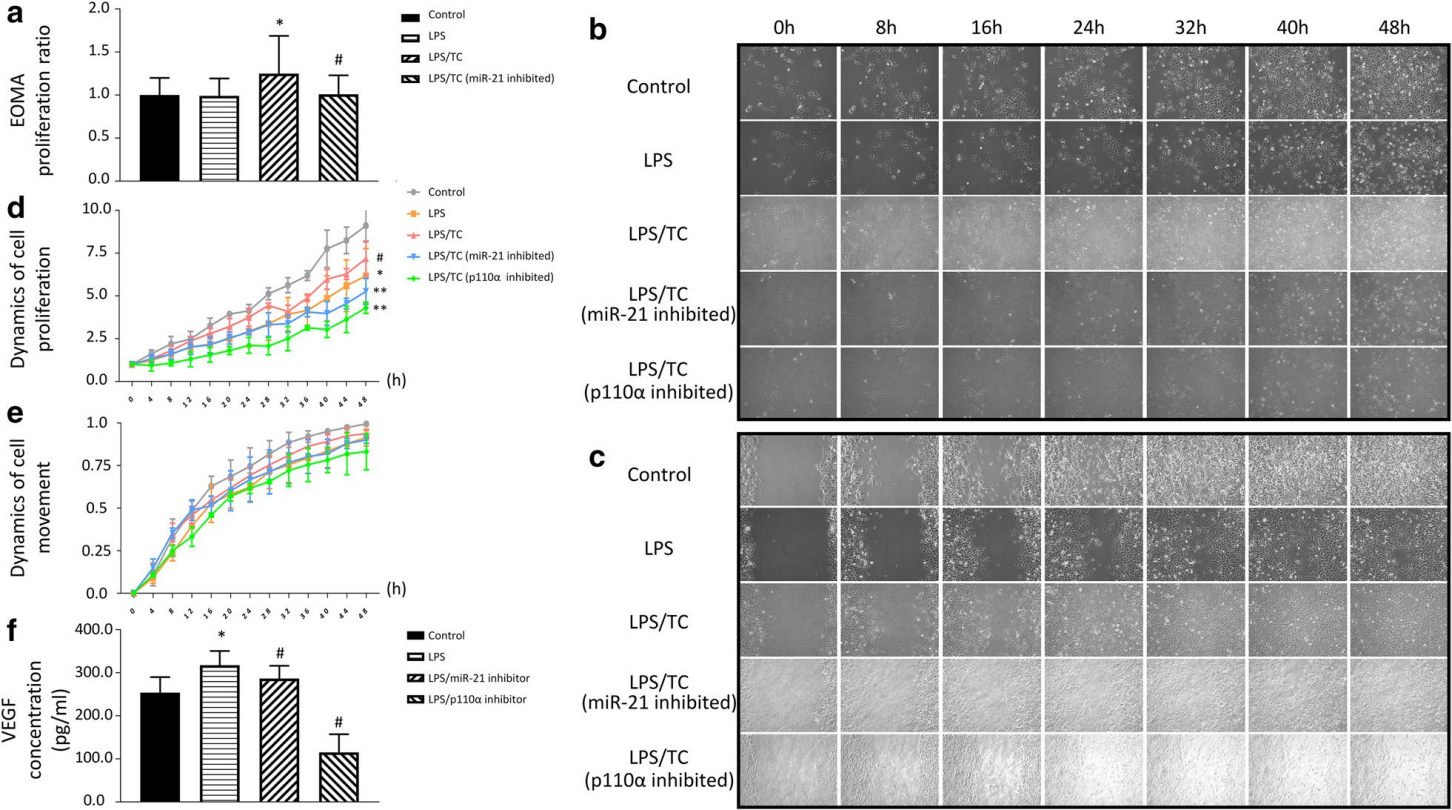
MiR-21a-3p and p110α mediated the promotion of TCs on EOMA proliferation induced by LPS. **a** Cells proliferation rate of EOMA treated with LPS and/or TCs and miR-21a-3p inhibitor measured by CCK8 assay. *P < 0.05 vs Control, ^#^*P* < 0.05 vs LPS/TC. **b**, **d** Cell proliferation of EOMA treated with LPS and/or TCs and miR-21a-3p inhibitor or p110α inhibitor measured by Cell-IQ. *P < 0.05 vs Control, ^#^*P* < 0.05 vs LPS, **P < 0.05 vs LPS/TC, n = 6. **c**, **e** Cell movement of EOMA treated with LPS and/or TCs and miR-21a-3p inhibitor or p110α inhibitor measured by Cell-IQ. **f** VEGFA levels secreted by TCs treated with LPS and/or miR-21a-3p inhibitor or p110α inhibitor measured by ELISA. *P < 0.05 vs Control, ^#^*P* < 0.05 vs LPS, n = 6

The proliferation of EOMA cells was then estimated after co-culture with TCs pre-treated with the miR-21a-3p or PI3K p110α inhibitor. Culture medium from TCs stimulated with LPS promoted EOMA cells proliferation, as determined by the CCK8 assay. Compared with medium from NC TCs, culture medium from TCs with miR-21a-3p inhibition significantly reduced EOMA cells proliferation (Fig. 7a). The effect of p110α was examined by dynamic real-time cell observation. The proliferation assay indicated that EOMA cells proliferation decreased with LPS stimulation but was restored by co-culture with TCs. Inhibition of miR-21a-3p or p110α (with its inhibitor HS-173) weakened the protective effect of TCs (Fig. 7b, d). The scratch assay showed similar results (Fig. 7c, e). VEGF protein expression was significantly elevated with LPS stimulation, and this increase was reversed by inhibition of either miR-21a-3p or p110α (Fig. 7f). The results above indicated that VEGF is regulated by both miR-21a-3p and p110α.

## Discussion

**Fig. 8:**
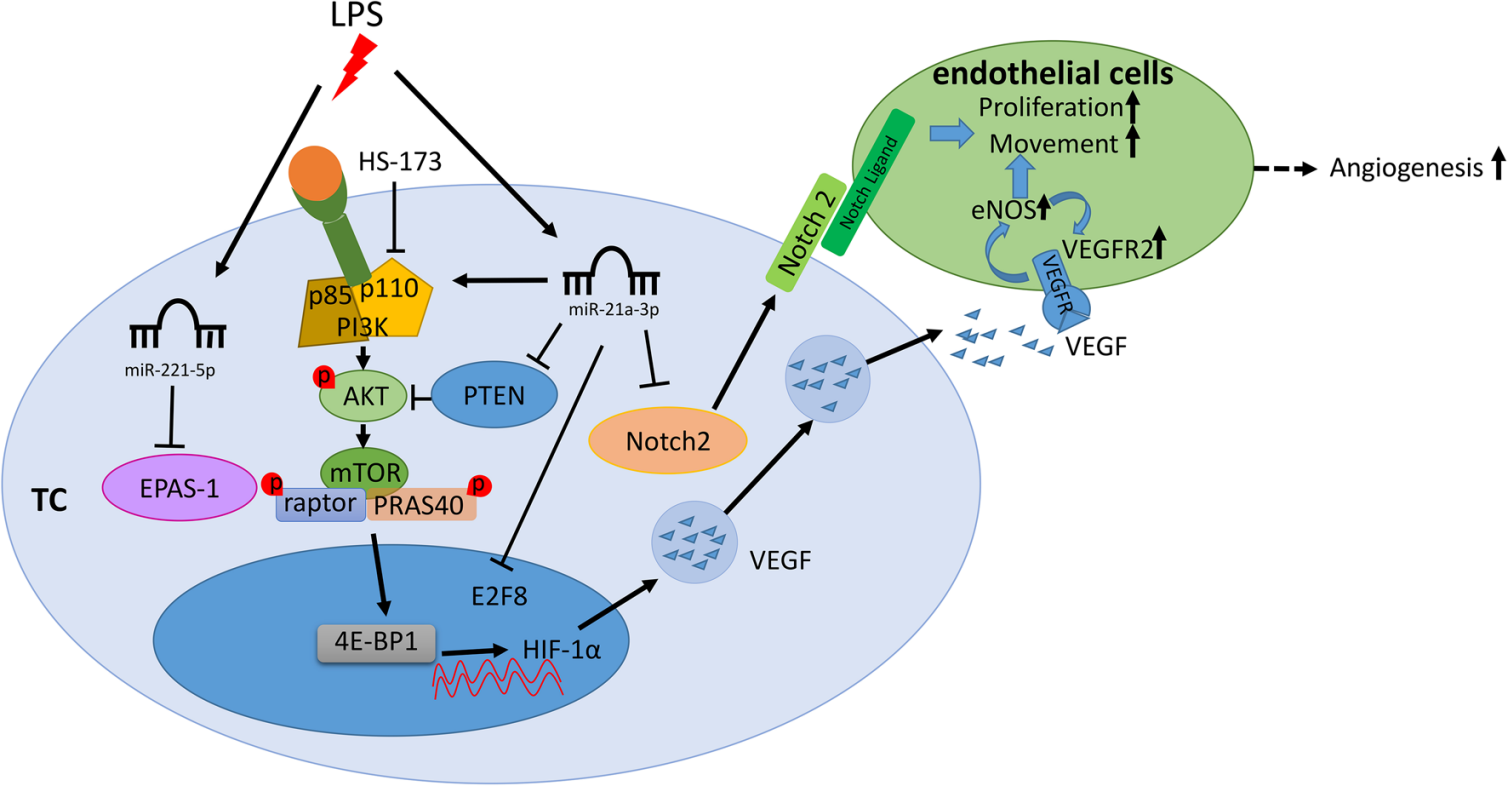
Schematic representation of the working model. TCs induced by LPS promoted endothelial regeneration and angiogenesis through miR-21a-3p-PI3K (p110α)/AKT/mTOR and VEGF signalling in TCs. The E2F8/Notch2 signalling pathway might also participates in this procession

This study reports that TCs culture medium can alleviate ARDS in mice probably via angiogenesis-associated factors regulated by miR-21a-3p. TCs exposed to LPS exhibited increased miR-21a-3p expression and VEGF production, which further promoted vascular endothelial cell proliferation. The protective effects of TCs mediated by miR-21a-3p might be regulated through PI3K (p110α)/AKT/mTOR signalling and the expression levels of the downstream targets E2F8 and Notch 2 (Fig. 8).

Endotoxin-induced ARDS has been reported to affect both respiratory epithelial cells and the underlying vascular endothelial cells [34]. In the present study, LPS stimulation induced severe vascular damage in the lungs, as shown by the reduced levels of CD31 and eNOS. TCs are distinct from mesenchymal stem cells and fibroblasts and have been reported to have specific roles in cell signalling, tissue remodelling and angiogenesis [35]. In the present study, TCs culture medium exhibited great potential to reverse the angiogenic signalling that was reduced by LPS-induced inflammation, supporting the observation that TCs alleviate LPS-induced lung injury in mice by releasing angiogenic factors [11].

Non-coding miRNAs are involved in several pathological processes, including angiogenesis [36, 37]. MiR-221-5p [38], miR-146a-5p [39], and miR-21a-3p [40–42] are reported to be associated with the angiogenesis process. MiR-21a-3p and miR-221-5p were demonstrated to be involved in the promotion of angiogenesis in TCs. As miR-21a-3p was more frequently reported on angiogenesis, it was further studied. MiR-21a-3p knockdown in TCs reduced CD31 and eNOS expression in the lungs of ARDS mice in vivo. MiR-21a-3p exerts its protective effects on injury repair by inducing angiogenesis-associated signalling pathways. For instance, miR-21a-3p activates the AKT pathway and increases matrix metalloproteinase-2 (MMP-2) expression to reduce the extent of the infarcted region in heart ischaemia/reperfusion injury [41], inhibits PTEN and sprouty homolog 1 (SPRY1) to heal soft tissue wounds [40], and upregulates VEGF and activates the Ang-1/Tie-2 axis in traumatic brain injury [42]. In the current study, the p110α isoform in PI3K/AKT/mTOR signalling pathway was demonstrated to be involved in miR-21a-3p-mediated angiogenic factor induction in TCs. However, the alteration of other protein levels and HIF-1α in TCs treated with LPS and the miR-21a-3p inhibitor indicated that more complex signalling pathways were involved in regulating the angiogenic function of TCs. Culture medium from LPS-induced TCs promoted EOMA cells proliferation in vitro, accompanied by elevated levels of VEGF mRNA and secretion, which further demonstrated that the functional miR-21a-3p was generated by TCs. These data support the hypothesis that miR-21a-3p plays a role in angiogenesis and profoundly demonstrate the mechanisms mediated by PI3K p110α.

The E2F family was first reported to induce cell proliferation [43], and E2F family members are essential transcriptional regulators of cell cycle progression [44], as well as apoptosis, metabolism and angiogenesis [45]. E2F8 is an atypical transcriptional repressor in the E2F family since it contains domains that differ from the canonical domains [46]. By forming homodimers or heterodimers with E2F7, E2F8 reduces the excessive and destructive activation of E2F1 [47]. However, reports of E2F8 in angiogenesis in the literature are controversial. E2F7/8 has been reported to positively regulate the formation of blood vessels during embryonic development via HIF-1α/VEGFA signalling [33]. On the other hand, E2F7/8 suppresses tumour angiogenesis via the induction of DLL4 [32]. In the present study, E2F8 expression was reduced after LPS stimulation in TCs and restored with miR-21a-3p inhibition, indicating that E2F8 plays a negative role in angiogenesis under inflammatory conditions.

The Notch family, which contains several receptors and ligands, is fundamental in the regulation of blood vessel branching [48]. DLL4, a Notch ligand, has an inhibitory function in blood vessel branching [49] that is compromised by Jagged 1 activation [50]. Notch1 positively regulates angiogenesis [51], while Notch2 negatively regulates cell proliferation [52] and angiogenesis [53]. In the initial stage of angiogenesis, inhibition of Notch 2 promotes vascular endothelial cell proliferation, while activation of Notch 2 reduces endothelial cell responses to VEGF [54, 55]. In the present study, Notch2 expression was mediated by miR-21a-3p. However, the relationship between the transcription factor E2F8 and Notch2 was not illustrated. Further experiments should be conducted to confirm the signalling pathway of E2F8/Notch2 in angiogenesis.

## Conclusion

TCs have been reported to be important in tissue repair and healing processes. Via mouse models, bioinformatics approaches and molecular biological methods, the present study shows that activated TCs promote endothelial regeneration and angiogenesis through miR-21a-3p-PI3K (p110α)/AKT/mTOR signalling and further demonstrates the key roles of VEGF in TCs. The E2F8/Notch2 signalling might also participates in this process. These findings shed light on miR-21a-3p in TCs as a new therapeutic target for vessel protection.

## Supplementary information

**Additional file 1: Figure S1.** The morphology of TCs. The pictures were gathered by Cell-IQ every 8 h. The white arrow showed the typical telopode.

## Data Availability

Not applicable.

## Abbreviations

ARDS: acute respiratory distress syndrome
TC: telocyte
PI3K: phosphoinositide-3-kinase
RTK: receptor tyrosine kinase
EGFR: epidermal growth factor receptor
FGFR: fibroblast growth factor receptor
VEGF: vascular endothelial growth factor
mTOR: mammalian target of rapamycin
IFN-γ: interferon-γ
miRNAs: microRNA
NSCLC: non-small cell lung cancer
LPS: lipopolysaccharide
CAD: chronic renal allograft dysfunction
SV40: simian vacuolating virus 40
TGF-β: transforming growth factor-β
EOMA cells: hemangioendothelioma endothelial cells
NC: negative control
DMEM/F12: Dulbecco’s modified Eagle’s medium/F12
FBS: foetal bovine serum
DAVID: Database for Annotation, Visualization and Integrated Discovery
cDNA: complementary DNA
qPCR: quantitative real-time polymerase chain reaction
HIF-1α: hypoxia inducible factor-1α
PMSF: phenylmethylsulfonyl fluoride
DLL4: Delta-like 4
PTEN: phosphatase and tension homolog on chromosome ten
ELISA: enzyme-linked immunosorbent assay
HE: hematoxylin–eosin
eNOS: endothelial nitric oxide synthase
DAPI: 4′,6-diamidino-2-phenylindole
ANOVA: analysis of variance
GO: gene ontology
SPRY1: sprouty homolog 1
